# Complete Mitochondrial Genome of the Free-Living Earwig, *Challia fletcheri* (Dermaptera: Pygidicranidae) and Phylogeny of Polyneoptera

**DOI:** 10.1371/journal.pone.0042056

**Published:** 2012-08-06

**Authors:** Xinlong Wan, Man Il Kim, Min Jee Kim, Iksoo Kim

**Affiliations:** 1 College of Agriculture & Life Sciences, Chonnam National University, Gwangju, Republic of Korea; 2 Western District Office, National Forensic Service, Jangseong-gun, Jeonnam-do Province, Republic of Korea; University of Veterinary Medicine Hanover, Germany

## Abstract

The insect order Dermaptera, belonging to Polyneoptera, includes ∼2,000 extant species, but no dermapteran mitochondrial genome has been sequenced. We sequenced the complete mitochondrial genome of the free-living earwig, *Challia fletcheri*, compared its genomic features to other available mitochondrial sequences from polyneopterous insects. In addition, the Dermaptera, together with the other known polyneopteran mitochondrial genome sequences (protein coding, ribosomal RNA, and transfer RNA genes), were employed to understand the phylogeny of Polyneoptera, one of the least resolved insect phylogenies, with emphasis on the placement of Dermaptera. The complete mitochondrial genome of *C. fletcheri* presents the following several unusual features: the longest size in insects is 20,456 bp; it harbors the largest tandem repeat units (TRU) among insects; it displays T- and G-skewness on the major strand and A- and C-skewness on the minor strand, which is a reversal of the general pattern found in most insect mitochondrial genomes, and it possesses a unique gene arrangement characterized by a series of gene translocations and/or inversions. The reversal pattern of skewness is explained in terms of inversion of replication origin. All phylogenetic analyses consistently placed Dermaptera as the sister to Plecoptera, leaving them as the most basal lineage of Polyneoptera or sister to Ephemeroptera, and placed Odonata consistently as the most basal lineage of the Pterygota.

## Introduction

Typical metazoan mitochondrial DNA (mtDNA) encodes for 13 proteins, 22 tRNAs, 2 rRNAs, and harbors a single non-coding control region that regulates the transcription and replication of the mtDNA [1], [2]. The size of the insect mitochondrial genome ranges from 14 to 19 kb [2], [3]; one exception is the 30–36 kb-long heteroplasmic genome of the bark weevil, which was determined by size estimation [4]. The source of the size variation is mainly the control region, and is due to the presence of variable lengths and numbers of tandem repeats [5], [6]. Another source is the presence of large intergenic spacers apart from the control region, an infrequent occurance in insects [4], [7]–[9]. These are composed mainly of a variable number of tandem repeats (e.g., a 1,448-bp long intergenic spacer composed of seven 202-bp tandem repeats and a partial 99-bp copy as found in *Acyrthosiphon pisum*; GenBank: FJ411411) or a non-repetitive sequence of substantial length (e.g., a 733-bp long non-coding sequence as found in *Tetraleurodes acacia* [9]). Either or both of these two types of large intergenic spacers substantially contribute to the whole genome size in some insects.

Because gene rearrangement is an important evolutionary event in the mitochondrial genome, it likely is regarded as a phylogenetic signal for the inference of evolutionary relationships, e.g. in bilaterian phylogeny [10]. Currently, 11 of 25 insect orders have shown mitochondrial gene rearrangement from the ancestral arrangement of insects, which is identical to that of *Drosophila* [11], [12]. However, among the 60 sequenced polyneopteran insects representing 9 orders, rearrangement is limited only to the Orthoptera where it involves 2 translocated tRNAs [13] and the Embioptera which shows substantial gene rearrangements [14]. Nevertheless, considering that full-length mitochondrial genome information for polyneopteran Dermaptera and Zoraptera is still unavailable, estimation of the magnitude and extension of gene rearrangement in this group remains far from completion.

Dermaptera are nocturnal insects with the characteristic feature of forceps-like, unsegmented cerci in adults that assists in predation and mating [15]. Dermaptera is divided into 3 extant suborders (Arixeniina, Forficulina, and Hemimerina) and one extinct suborder (Archidermaptera), with ∼ 2,000 extant species [16]. The history of Dermaptera dates back to the Late Triassic to Early Jurassic period of ∼208 million years ago (MYA), based on fossil records found in England and Australia [17]. It has been assumed that Dermaptera originated from Protelytroptera, which resembles modern Blattodea or cockroaches, but no fossil evidence of morphological change from Protelytroptera to Dermaptera has yet been unearthed [17]. The Grylloblattodea was once regarded as very closely related to Dermaptera [18], although a closer relationship of Phasmatodea, Embioptera, Plecoptera, or Dictyoptera to Dermaptera has also been posited [19].

Until now, diverse morphological characteristics [20]–[23] and molecular data from nuclear and mitochondrial genes have been employed to infer phylogenetic relationships among the polyneopteran orders [24]–[27]. From the morphological perspective, Hennig [20] and Wheeler et al. [28] placed Dermaptera as the sister to Dictyoptera on the basis of synapomorphic wing elevators and a basisternal fold, but Kukalová-Peck [22] obtained unresolved relationships among Dermaptera, Grylloblattodea, and Dictyoptera, placing this clade as the sister to the remainders of the Polyneoptera, based on comprehensive morphological data including wing and mouthpart structures. In terms of molecular data, 18S rDNA suggested that Dermaptera was the sister to Plecoptera as a basal lineage for Polyneoptera [26]. However, combined molecular and morphological data instead supports the placement of Dermaptera in an unresolved clade including Grylloblattodea, Zoraptera, and Dictyoptera [28] or a sister relationship between Dermaptera and Zoraptera [25].

In this study, we sequenced the complete mitochondrial genome of *Challia fletcheri*: the first complete mitochondrial genome for Dermaptera. Although its distribution includes Korea, Japan, and China, this species is listed as an endangered animal in Korea due to its annual decrease in numbers [29], [30]. The genomic sequence of the species is herein described and compared to other polyneopteran insects in terms of comparative genomics. In addition, the genomic sequence was employed for the inference of the phylogenetic position of Dermaptera among the polyenopteran orders.

## Results and Discussion

### General Features of the Genome

**Figure 1:**
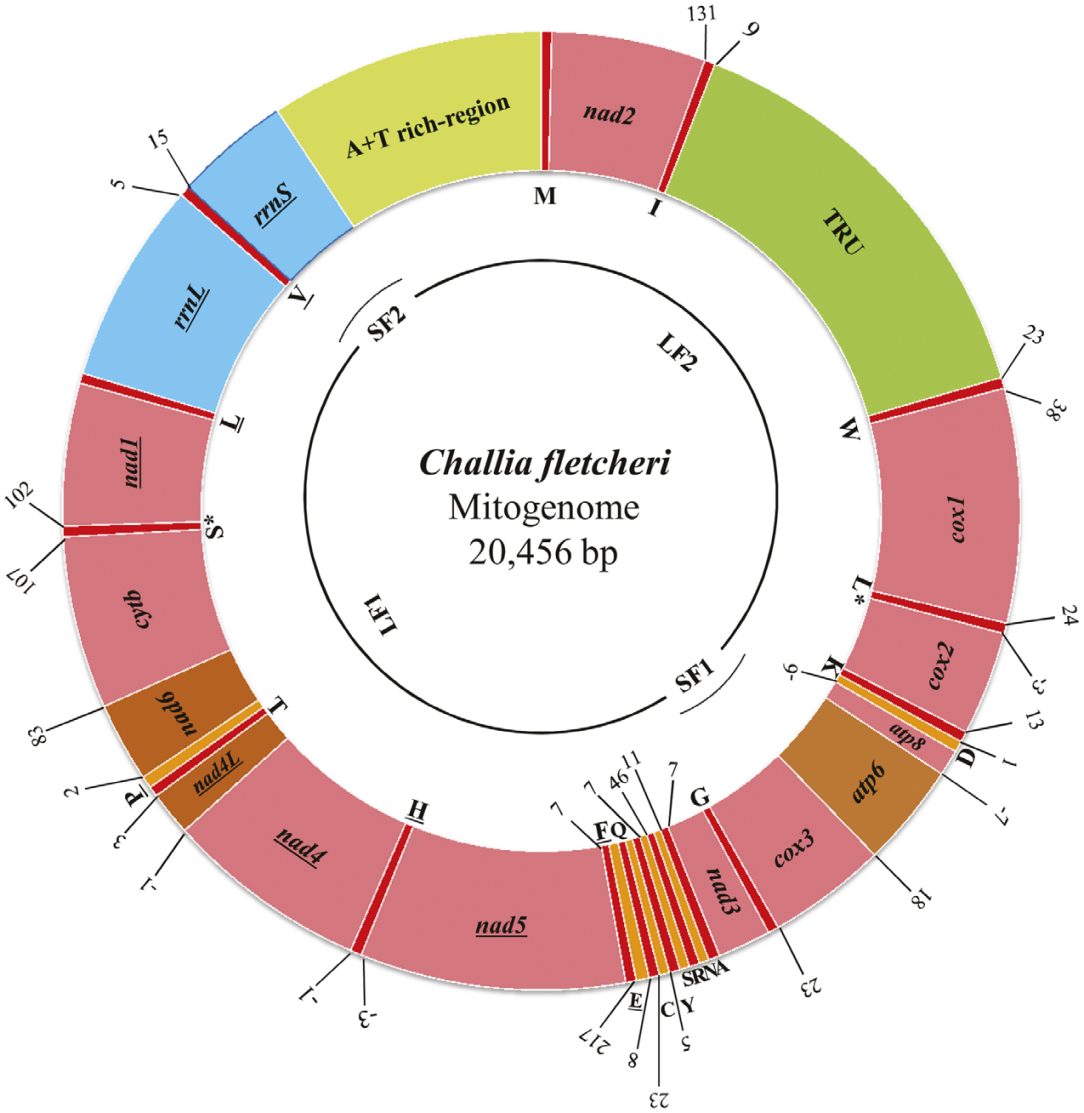
Circular map of the mitochondrial genome of *C. fletcheri.* tRNAs are denoted as one-letter symbols consistent with the IUPAC/IUB single letter codes for amino acids, with L  =  *trnL*(CUN); L*  =  *trnL*(UUR); S  =  *trnS*(AGN); S*  =  *trnS*(UCN). TRU indicates the tandem repeat unit. Gene names that are not underlined indicate a clockwise transcriptional direction, whereas underlines indicate a counter-clockwise transcriptional direction. Numbers show the sizes of intergenic spacers (positive values) and overlapping region between genes (negative values). The *C. fletcheri* mitochondrial genome was sequenced by 2 overlapping short (SF1 and SF2) and long (LF1 and LF2) fragments, as shown in a single line within the circle.

The complete mitochondrial genome of *C. fletcheri* (GenBank: JN651407) is 20,456-bp in size (Figure 1; Table 1). This is the largest size among the insect mitochondrial genomes sequenced so far, although a bark weevil mitochondrial genome has been estimated to be 30–36 kb [4]. Such a long mitochondrial genome is mainly attributed to the expansions of large non-coding regions and the A+T-rich region. The *C. fletcheri* contains the ancestral 37 genes, 9 PCGs and 17 tRNAs encoded in the major strand, and 4 PCGs, 5 tRNAs, and 2 rRNAs encoded in the minor strand (Figure 1).

**Table 1:** Summary of mitochondrial genome of *C. fletcheri*.

Gene	Direction	Nucleotide number	Size	Anticodon	Start codon	Stop codon
*trnM*	F	1–68	68	31–33 CAT	–	–
*nad2*	F	69–1085	1017	–	ATT	TAA
*trnI*	F	1217–1285	69	1247–1249 GAT		
TRU		1295–4150	2856	–		
*trnW*	F	4174–4245	72	4206–4208 TCA	–	–
*cox1*	F	4284–5825	1542	–	ATA	TAA
*trnL*(UUR)	F	5850–5920	71	5884–5886 TAA	–	–
*cox2*	F	5918–6601	684	–	ATG	TAA
*trnK*	F	6615–6685	71	6645–6647 CTT	–	–
*trnD*	F	6687–6760	74	6720–6722 GTC	–	–
*atp8*	F	6755–6925	171	–	ATA	TAG
*atp6*	F	6919–7602	684	–	ATG	TAA
*cox3*	F	7621–8406	786	–	ATG	TAG
*trnG*	F	8430–8500	69	8463–8465 TCC	–	–
*nad3*	F	8501–8854	354	–	ATT	TAG
*trnA*	F	8862–8935	74	8892–8894 TGC	–	–
*trnN*	F	8947–9011	65	8978–8980 GTT	–	–
*trnR*	F	9012–9078	67	9043–9045 TCG	–	–
*trnS*(AGN)	F	9125–9191	67	9150–9152 GCT	–	–
*trnY*	F	9199–9268	70	9228–9230 GTA		
*trnC*	F	9274–9340	67	9303–9305 GCA	–	–
*trnQ*	F	9364–9432	69	9394–9396 TTG	–	–
*trnE*	R	9441–9526	86	9494–9496 TTC	–	–
*trnF*	R	9744–9812	69	9774–9776 GAA	–	–
*nad5*	R	9819–11570	1752	–	ATC	TAA
*trnH*	R	11568–11641	74	11597–11599 GTG	–	–
*nad4*	R	11641–12981	1341	–	ATG	TAA
*nad4L*	R	12981–13271	291	–	ATA	TAA
*trnT*	F	13275–13341	67	13308–13310 TGT	–	–
*trnP*	R	13342–13422	81	13388–13390 TGG	–	–
*nad6*	F	13425–13949	525	–	ATT	TAA
*cytb*	F	14033–15169	1137	–	ATG	TAG
*trnS*(UCN)	F	15277–15347	71	15307–15309 TGA	–	–
*nad1*	R	15450–16388	939	–	ATG	TAA
*trnL*(CUN)	R	16389–16454	66	6422–16424 TAG	–	–
*rrnL*	R	16455–17788	1334	–	–	–
*trnV*	R	17794–17862	69	17825–17827 TAC	–	–
*rrnS*	R	17878–18640	763	–	–	–
A+T-rich region		18641–20456	1816	–	–	–

The nucleotide composition of the *C. fletcheri* mitochondrial genome is biased toward A/T nucleotides, which account for 72.5%, and this value is well within the range detected in mitochondrial genomes of Polyneoptera (Table S1). In terms of individual nucleotides, the compositions of A, T, and C in *C. fletcheri* are 28.2%, 40.3%, and 13.9%, respectively, and these values are well within the ranges found in Polyneoptera. However, the G content in *C. fletcheri* (17.6%) is slightly higher than in any other species (Table S2). The codon-based nucleotide composition of *C. fletcheri* indicates that the A/T content at the third codon position (79.3%) is higher than that of the second and first codon positions (63.9% and 62.5%, respectively) (Table S2).

All *C. fletcheri* PCGs start with typical invertebrate initiation codons; either isoleucine (*cox1*, *atp8*, *nad2*, *nad3*, *nad4L*, *nad5*, and *nad6*) or methionine (*cox2*, *cox3*, *atp6*, *cytb*, *nad1*, and *nad4*), and end with the complete termination codon, TAG (4 genes) or TAA (9 genes) (Table 1). Among 13 PCGs, 4 are neighbors to another PCG: *atp8*-*atp6*, *atp6*-*cox3*, *nad4*-*nad4L*, and *nad6*-*cytb*. These are either interspaced (*atp6*-*cox3* and *nad6*-*cytb*) or overlapping (*atp8*-*atp6* and *nad4*-*nad4L*) (Table 1). The secondary structure formed by transcribed polycistronic mRNA has been proposed to be responsible for cleavage of such neighboring proteins, which is analogous to the tRNA excision model [12], [31]. However, a more recent investigation has shown the importance of a base-pair mismatch, which causes a bulge located close to the start codon of a downstream protein in the stem-and-loop structure. The bulge was suggested to provide a physical attribute for the enzyme to recognize the cleavage site at the start codon of the downstream protein [32].

**Figure 2:**
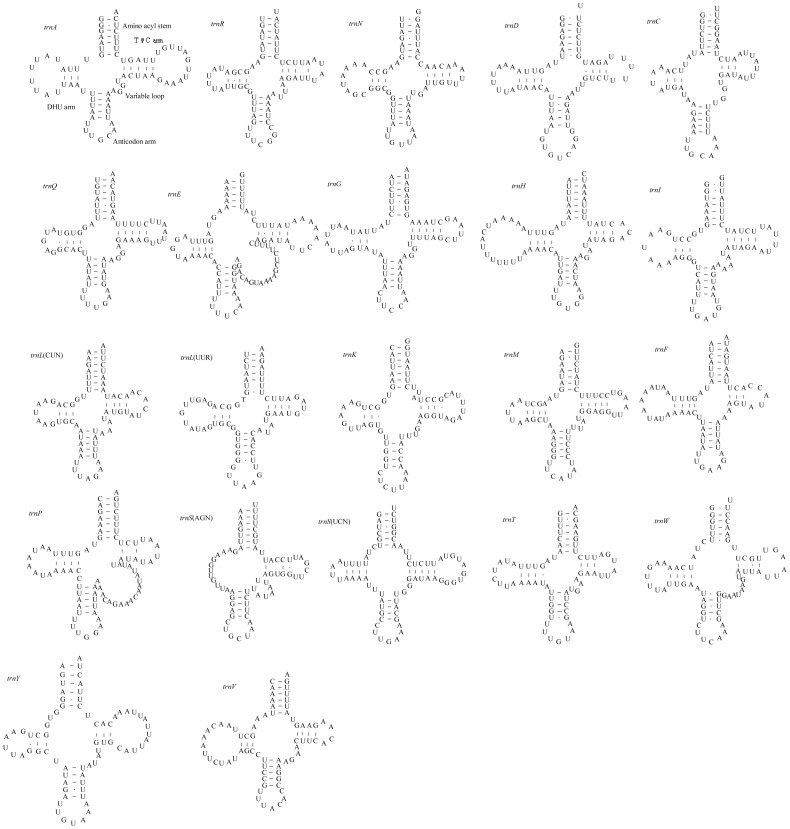
Predicted secondary structures for the 22 tRNA genes of *C. fletcheri.* Dashes (–) indicate Watson-Crick base-pairing and centered dots (•) indicate G-U base-pairing. Arms of tRNAs (clockwise from top) are the amino acid acceptor (AA) arm, TψC (T) arm, the anticodon (AC) arm, and the dihydrouridine (DHU) arm.

The complete set of 22 tRNAs (one specific for each amino acid, and 2 each for leucine and serine) found in metazoan mitochondrial genomes were identified in *C. fletcheri*, and they are interspersed throughout the genome (Table 1; Figure 2). Of the 22 tRNAs, 21 can be folded into a typical cloverleaf structure with a 7-bp amino-acyl stem, 5-bp anticodon arm, 7-bp anticodon loop, a variable loop, DHU arm, and a TψC arm. Unlike others, the *trnS*(AGN) lacks the DHU stem, and instead, has a 12-bp long DHU loop, as is commonly found in metazoan mitochondrial genomes [3]. A somewhat unusual feature found in *trnE* and *trnP* is the presence of much larger variable loops (20 and 19-bp long, respectively; Figure 2) than in other insects. The anticodons of *C. fletcheri* tRNAs (Table 1) are all identical to those of the respective tRNAs found in the other Polyneoptera (data not shown).

The *rrnL* and *rrnS* genes are located between *trnL* (CUN) and *trnV*, and between *trnV* and the A+T-rich region, respectively. The lengths of *rrnL* and *rrnS* are 1,334 bp and 763 bp, and A/T contents are 70.9% and 70.2%, respectively (Table S1). These values are well within the ranges found in Polyneoptera.

**Figure 3:**
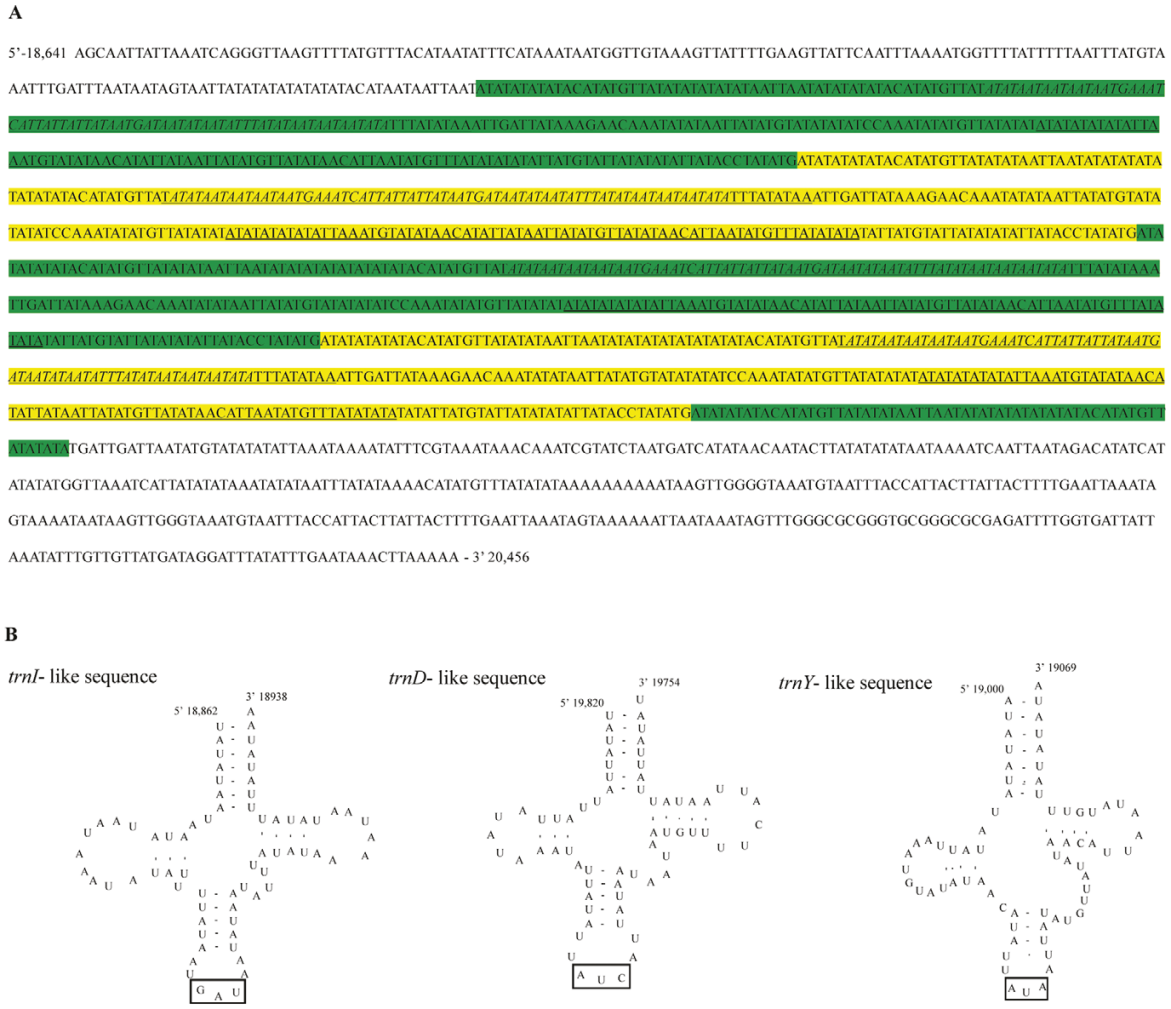
Tandem repeat units and tRNA-like sequences found in the A+T-rich region of *C. fletcheri*. (A) The A+T-rich region sequence of *C. fletcheri*; (B) Predicted secondary structures for 3 tRNA-like sequences found in the A+T-rich region. The sequences covered by green and yellow are tandem repeat units; the single underline and double underlines indicate *trnI*-like and *trnY*-like sequences, respectively; the italic nucleotides indicate *trnD*-like sequences; the rectangular boxes indicate the respective anticodons, and the nucleotide position is indicated at the beginning and end sites of the sequence.

The A+T-rich region of *C. fletcheri* is 1,816 bp long, is located between *rrnS* and *trnM*, and contains a high A/T content of 89.31% (Table S1). Such a long *C. fletcheri* A+T-rich region is attributable to 4 tandem repeats composed of 2 identical 297-bp copies, 2 nearly identical copies, plus a 60-bp partial copy of the beginning of the repeat (Figure 3A). Along with the tandem repeats, each of the 4 identical *trnY*-like, *trnI*-like, and *trnD*-like sequences were detected (Figures 3A and 3B). The sequence homology between the regular and tRNA-like sequence was 50% for *trnY*, 66% for *trnI*, and 59% for *trnD*, showing substantial sequence divergence between them. The long tandem repeats also have been found in several other polyneopteran A+T-rich regions, such as Mantodea, Isoptera, and Orthoptera [33]–[35]. For example, the *Reticulitermes hageni* (Isoptera) A+T-rich region harbored two 189-bp tandem repeats plus an 89-bp partial copy of the beginning of the repeat, and two 554-bp tandem repeats plus a 99-bp partial copy of the beginning of the repeat [34].

A total of 11 different stem-and-loop structures were detected on the major strand of the *C. fletcheri* A+T-rich region and 2 of them harbor the conserved flanking sequences TATA and G(A)_n_T at the 5′ and 3′ end, respectively (Figure S1). The stem-and-loop structure on the minor strand of the A+T-rich region was thought to be associated with the replication origin of the major strand, and the conserved flanking sequences at each end of the structure have regulatory roles for recognition of the replication origin [36]. However, more recently, Saito et al. [37] proposed that the poly-T stretch located on the minor strand near the *trnI* in holometabolous insects is involved in replication initiation of mtDNA. A 10-bp long analogous poly-T stretch (with one A insertion) was detected in the *C. fletcheri* A+T-rich region, but it is located near the 5′ end of the *rrnS* gene on the major strand. As for some other hemimetabolous insects (e.g., Orthoptera) which do not possess an obvious poly-T stretch in the A+T-rich region, the stem-and-loop structures were suggested to perform the role of the poly-T stretch [37].

### Non-coding Regions

**Figure 4:**
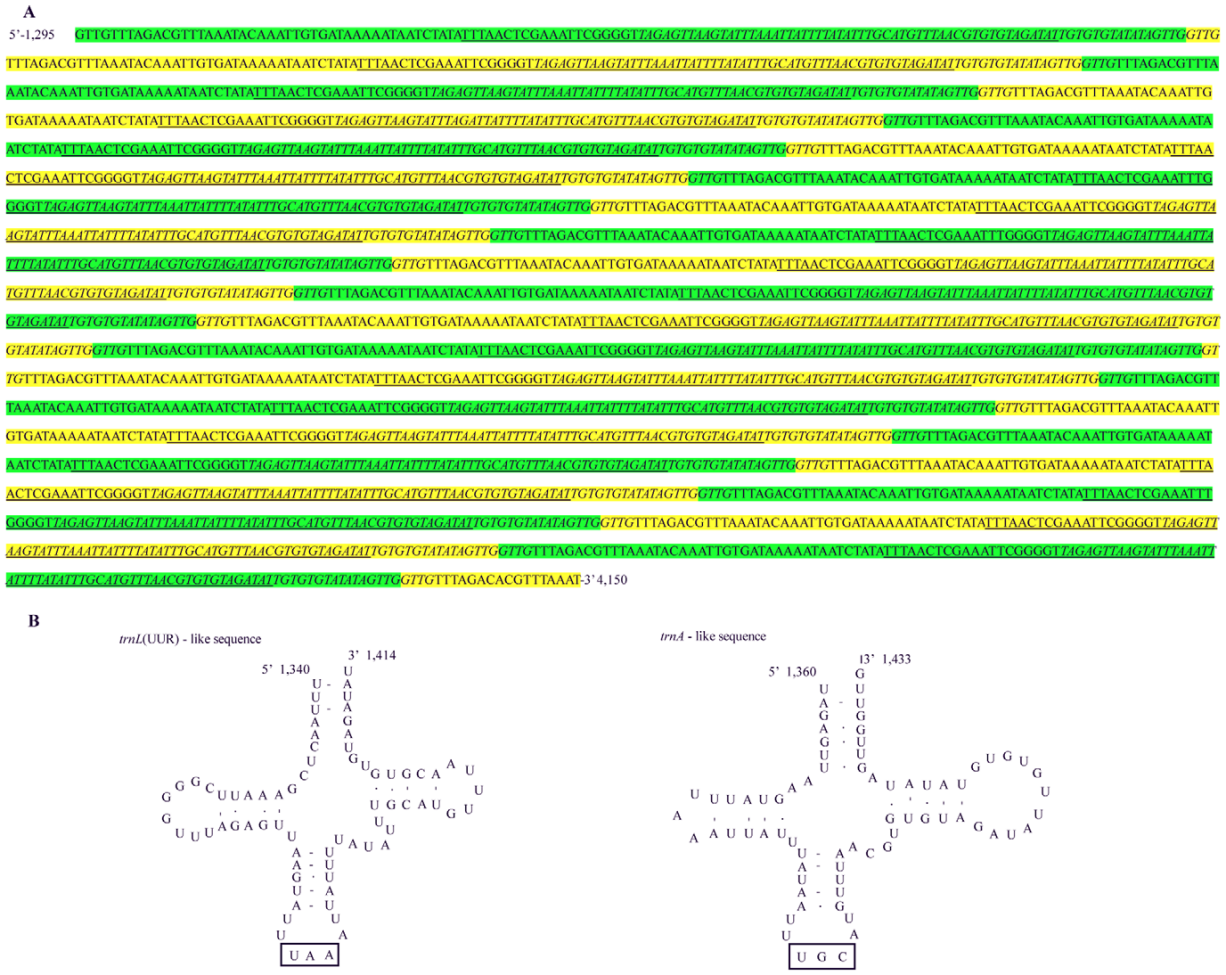
Tandem repeat units (TRU) and secondary structures of *trnL*(UUR)-like and *trnA*-like sequences found in the TRU. (A) TRU; (B) The secondary structures of *trnL*(UUR)-like and *trnA*-like sequences found in the TRU. The sequences covered by yellow and green indicate each repeat unit; the underlined sequences indicate *trnL*(UUR)-like sequences; the italic nucleotides indicate *trnA*-like sequences; the rectangular boxes indicate the respective anticodons, and the nucleotide position is indicated at the beginning and end sites of the sequence.

Except for the A+T-rich region, a total of 24 non-coding regions ranging in size from 1 to 2,888 bp (a total of 3,784 bp) were interspersed throughout the *C. fletcheri* mitochondrial genome (Figure 1), and the total length is the longest in Polyneoptera (data not shown). Among these non-coding regions, the longest one (2,888 bp) is located between *trnI* and *trnW*, spanning a 2,876 bp tandem repeat unit (TRU) without coding any protein. This TRU consists of twenty-one 135-bp tandem repeats plus a 21-bp partial copy of the beginning of the repeat (Figure 4A). Within the TRU, 20 identical *trnL*(UUR)-like and 21 identical *trnA*-like sequences, along with the repeats, were identified by tRNA structure search. The sequence similarity between the regular and tRNA-like sequence was 51% for *trnL*(UUR) and 60% for *trnA*. These tRNA-like sequences fold into cloverleaf structures harboring the corresponding anticodons (Figure 4B), but their functionality remains unknown. The 75.60% A/T content in the TRU is higher than that of the whole genome (13 PCGs, *rrnL*, and *rrnS*), but lower than that of the A+T-rich region (Table S1). To our knowledge, the size of this TRU is the longest reported in insects to date. In Hemiptera, a 1,513-bp long TRU located between *trnE* and *trnF* was found in the *A. pisum* mitochondrial genome (Unpublished, GenBank: FJ411411). This TRU contains seven 202-bp tandem repeats with the first repeat unit overlapping with the 3′ end of *trnE*, and a partial 99-bp copy of the beginning of the repeat. Similarly, a 1,724-bp long TRU has also been reported from the coleopteran, *Pyrocoelia rufa* [8]. In addition, a 409-bp long TRU including 4 identical *trnL*(UUR), which all were presumed to be functional was reported in the hymenopteran *Abispa ephippium* [38]. However, a similar TRU has never been found in the other mitochondrial genomes of Polyneoptera. Such TRUs in animal mitochondrial genomes could originate from slipped-strand mispairing, resulting in expanded repeat caused by an unequal crossing-over event due to the mispairing propensity of the simple tandem repeats [39].

**Figure 5:**
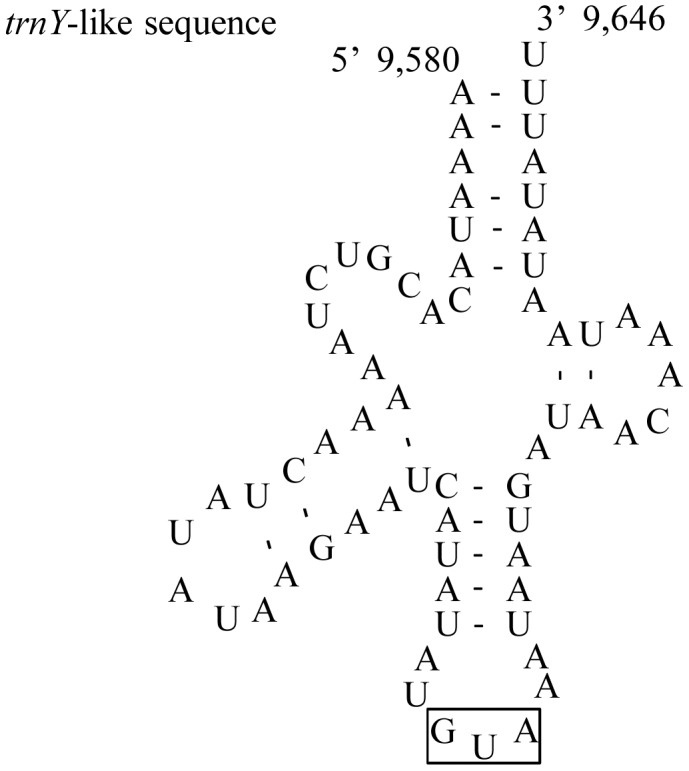
Predicted secondary structure for *trnY*-like sequence found in intergenic spacer between *trnE* and *trnF*. The rectangular boxes indicate the respective anticodons, and the nucleotide position is indicated at the beginning and end sites of the structures.

The second longest non-coding sequence (217 bp) is located between *trnE* and *trnF* (Figure 1), wherein 1 *trnY*-like sequence with a proper secondary structure and anticodon is found (Figure 5). This region is not homologous to any other gene or region of the genome. The sequence similarity between *trnY*-like and regular *trnY* gene is 58%. Another substantially large non-coding region (131 bp) is located between *nad2* and *trnI* and can be labeled as an unidentified opening reading frame (UORF) because it comprises a start codon (TTG) infrequently described in many insects and an incomplete stop codon (TA), encoding 43 amino acids. The encoded peptide, however, is not similar to any known sequence, although an extensive GenBank database on NCBI has been searched through BLAST (http://blast.ncbi.nlm.nih.gov/Blast.cgi). *Triatoma dimidiate* (Hemiptera) mitochondrial genome has been reported to have a UORF between *trnS*(UCN) and *nad1*, encoding 103 amino acids; the function of the UORF was presumed to be another origin of replication [7].

### Reversal Strand Asymmetry

Nucleotide bias, measured in terms of base skewness of AT and GC in the whole *C. fletcheri* genome (measured from major strand) is −0.166 and 0.346, respectively (Table S1), indicating that more Ts and Gs are encoded in the whole genome of *C. fletcheri*. All the other Polyneoptera have more As and Cs measured from the major strand. *C. fletcheri* is T- and G-skewed (−0.177 and 0.117, respectively) for concatenated PCGs. All other species of Polyneoptera harbor more Ts, but the G-skew is not unanimous among species in concatenated PCGs (Table S3). As for the PCGs of *C. fletcheri* encoded in the major strand (9 PCGs), the AT- and GC-skewness was found to be −0.327 and 0.383, respectively, whereas those in the minor strand were 0.059 and −0.315, respectively. In other species of Polyneoptera, the minor strand is T- and G-skewed, the reverse of *C. fletcheri*, but the major strand is either A and C-skewed or T- and C-skewed (Table 2; Table S3). This pattern of strand asymmetry has also been found in many animals, including arachnids and lepidopterans [40], [41].

**Table 2:** The number of species presenting AT- or GC-skewness in whole genome, whole PCGs, major strand, and minor strand PCGs in Polyneoptera.

Skewness	Categories			
	Whole genome	Whole PCGs	Major strand	Minor strand
A- and C-skewed	59	0	32	0
T- and G-skewed	0	12	0	59
T- and C-skewed	0	45	27	0
T-skewed*	0	2	0	0

* GC-skewness is zero.

Francino and Ochman [42] and Hassanin et al. [43] proposed that asymmetrical mutation pressure on the mitochondrial genome causes biased occurrence of mutations between strands. During replication of mtDNA, the parental minor strand remains as a single strand for a longer time until ∼ 97% of the nascent minor strand is replicated using the major strand as a template. Therefore, the parental minor strand is easily damaged by hydrolysis, resulting in the deaminations of A and C [43]–[45]. This results in A- and C-skewness on the major strand and T- and G-skewness on the minor strand. As described above, however, a substantial number of Polyneoptera evidence deviation from this general rule in the major strand, particularly in *C. fletcheri*, which shows a unique reversal in both major and minor strands. One of the plausible explanations for this is the inversion of replication origin located in the A+T-rich region [43], [44], resulting in the major strand being single-stranded for longer than the minor strand and resulting in A- and C-skewness on the minor strand and T- and G-skewness on the major strand [43]. By examination of the regulatory elements in the A+T-rich region, Wei et al. [46] found inverted A+T-rich regions in several insect species and the resultant reversal of strand asymmetry in the major strand. Their reasoning for inversion was based on the finding of inverted regulatory elements (e.g., the stem-and-loop structure with conserved flanking sequences at each end and poly-T stretch). The stem-and-loop structures found in the *C. fletcheri* A+T-rich region, are located in a reversed direction on the complementary strand compared to those generally found in other insects. Nevertheless, reverse strand asymmetry resulting from the inversion of the A+T-rich region may not be obvious if the event is recent [43], suggesting that the inversion event in *C. fletcheri* may have occurred a long ago.

### Mitochondrial Gene Rearrangement

**Figure 6:**
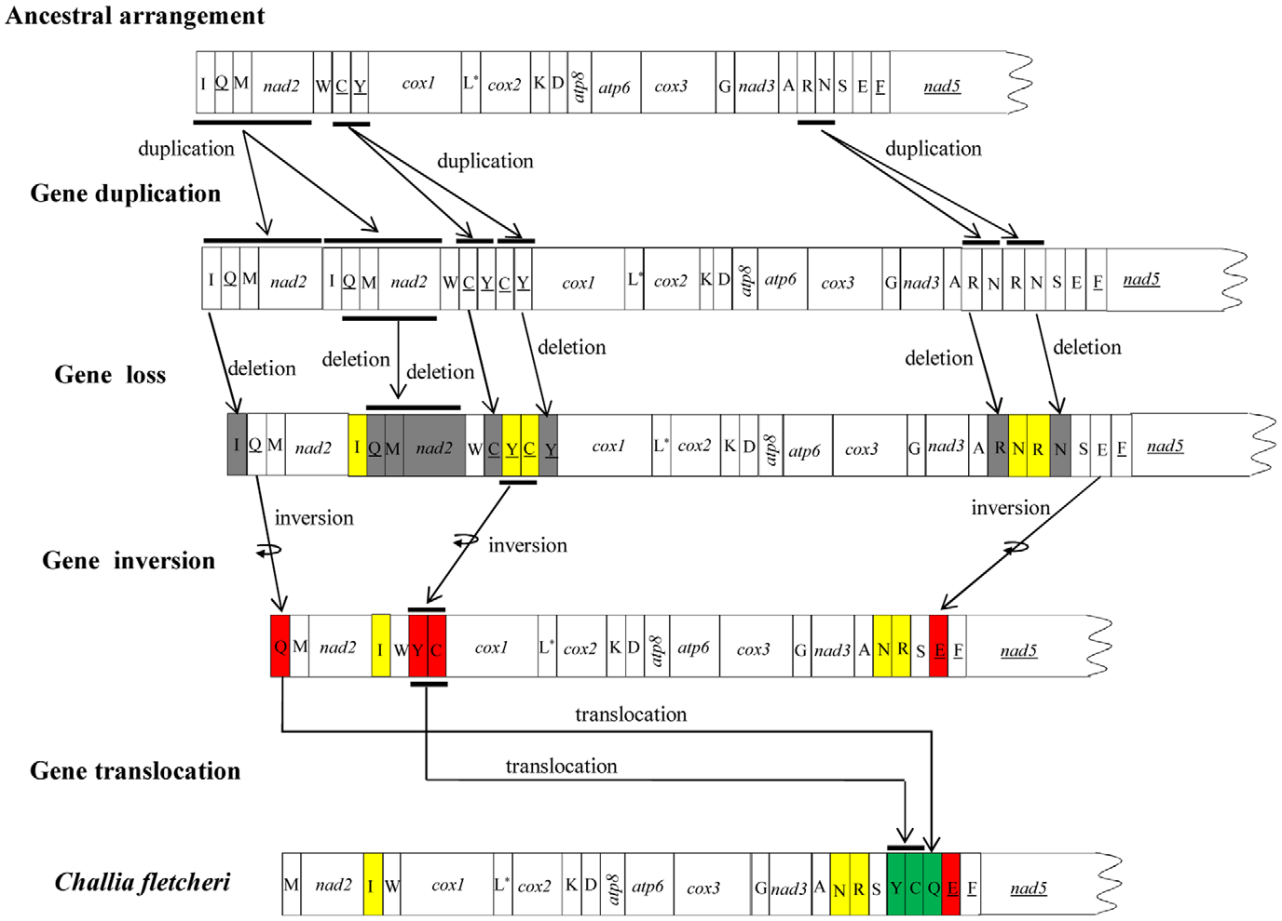
Schematic illustration of each event for mitochondrial gene rearrangement in *C. fletcheri.* Gene sizes are not drawn to scale. Gene names that are not underlined indicate a forward transcriptional direction, whereas underlines indicate a reverse transcriptional direction. tRNA genes are abbreviated using the one-letter amino acid code, with L*  =  *trnL*(UUR); S  =  *trnS*(AGN). White boxes represent genes with the same relative position as in the ancestral insect arrangement pattern. Yellow boxes represent gene translocations; red boxes represent gene inversions; green boxes represent gene shuffling with remote inversions compared to the ancestral insect arrangement. The grey boxes represent gene deletions. The remaining genes and gene orders identical to the ancestral insect are omitted. Each rearrangement event was independent, so there is no way of determining the order of event.

In contrast to the ancestral arrangement commonly found in insect mitochondrial genomes, *C. fletcheri* has a substantial number of tRNA rearrangements (Figure 6). These include 3 translocations resulting from 2 duplication/random deletion events (*trnI*, *trnN*, and *trnR*), 3 shuffling with remote inversions (*trnQ*, *trnY*, and *trnC*), and 1 local inversion (*trnE*). Several mechanisms responsible for mitochondrial gene rearrangement have been proposed: (1) translocation either by intramitochondrial recombination or by gene duplication/random loss, (2) inversion by intramitochondrial recombination, and (3) shuffling with remote inversion (translocation and inversion) either by combined intramitochondrial genome recombination and duplication/random loss of gene block, or by 2 separate intramitochondrial genome recombinations [47]–[51]. A schematic illustration of each possible event for *C. fletcheri* mitochondrial genome is presented in Figure 6. The translocation of *trnI* is probably caused by duplication of gene block *trnI-trnQ-trnM-nad2*, resulting in the arrangement *trnI-trnQ-trnM-nad2-trnI-trnQ-trnM-nad2*. A subsequent random loss of *trnI* in the first copy and *trnQ-trnM-nad2* in the second copy may have resulted in *trnQ-trnM-nad2*-*trnI*. Likewise, the duplication/random loss of the gene blocks *trnC*-*trnY* and *trnR*-*trnN* may also have resulted in an intermediate arrangement of *trnY*-*trnC* and the current arrangement of *trnN*-*trnR*, respectively (Figure 6). The local inversion of *trnE* may have been caused by the breakage of the mitochondrial genome at *trnE* and recombination of the *trnE* on the other strand at the same position [48], [52]. In the case of the shuffling with remote inversion of 3 tRNAs (*trnQ*, *trnY*, and *trnC*), this may be caused by 2 separate intramitochondrial recombinations. The first inversion event may include recombination of *trnQ* and the gene block *trnY*-*trnC* in opposite directions, and the second translocation event may be caused by recombination of *trnQ* and *trnY*-*trnC* in the same directions, resulting in shuffling with remote inversion. Nevertheless, as each rearrangement event was independent, there is no way of determining the actual order of events.

Boore [2] reported that the regions flanking the control region and between *nad3* and *nad5* are the hot spots for gene rearrangements in Arthropoda. In the *C. fletcheri* mitochondrial genome, most rearrangements occurred within these regions. For example, the rearranged *trnI* and *trnQ* are located in the region flanking the A+T-rich region in the ancestral arrangement, while the translocation of *trnR* and *trnN* and the local inversion of *trnE* occurred within the region between *nad3* and *nad5* (Figure 6).

All Polyneoptera except Orthoptera and Embioptera have shown mitochondrial gene arrangement identical to the ancestral type. In Orthoptera, 27 of 28 available species belonging to the suborder Caelifera have shown only 1 identical tRNA translocation, resulting in the order *trnD*-*trnK* instead of the ancestral order of *trnK*-*trnD*, and this rearrangement has been suggested to be synapomorphic for Acridomorpha [13]. In the case of another orthopteran suborder, Ensifera, 1 of 10 species has shown common translocation of 1 tRNA between *trnN* and *trnE* compared to the ancestral type (Figure S2). Owing to a paucity of rearrangements in Polyneoptera, it is difficult to speculate the possible mechanisms that might be responsible for the extensive mitochondrial genome rearrangement of *C. fletcheri*.

### Phylogeny of Polyneoptera

**Figure 7:**
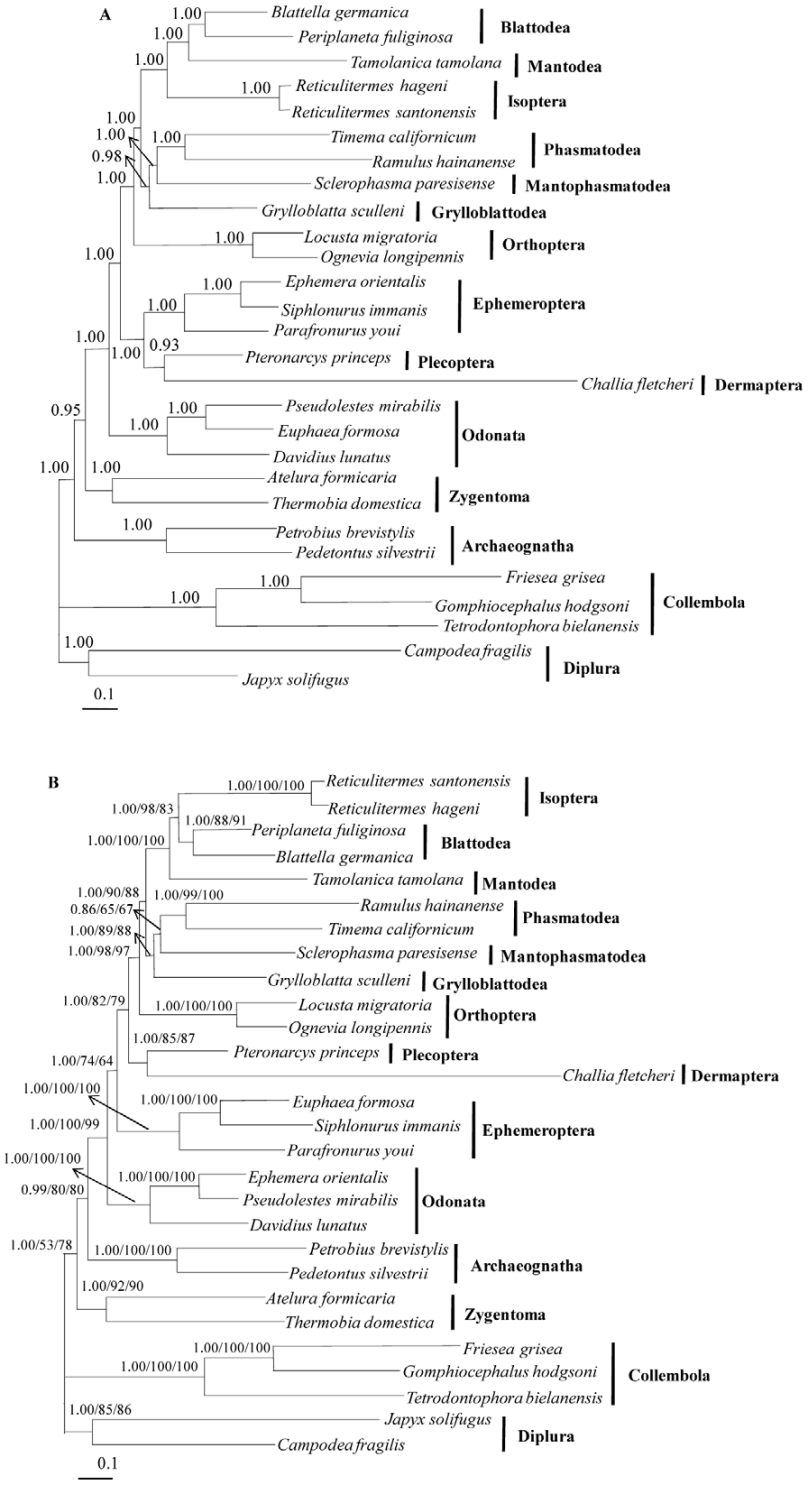
Phylogeny of polyneopteran orders. ( A) Bayesian inference phylogeny obtained with the dataset PCG123RNA; (B) Bayesian inference phylogeny obtained with the dataset PCG12RNA. The numbers associated with the nodes are posterior probabilities obtained by BI analysis with the dataset PCG12RNA (first) or bootstrap values obtained by ML analysis with the dataset PCG12RNA (second) and ML analysis with the dataset PCG123RNA (third). The species of Collembola and Diplura were utilized as outgroups to root the trees. The scale bar indicates the number of substitutions per site.

To study the phylogeny of Polyneoptera, we performed Bayesian Inference (BI) and Maximum Likelihood (ML) analyses based on several datasets such as all codon positions of 13 PCGs plus all RNAs (rRNAs and tRNAs) (PCG123RNA) and 1^st^ +2^nd^ codon positions of 13 PCGs plus all RNAs (PCG12RNA), along with 2 partitioning strategies for BI analyses. Here, we only present 2 phylogenetic topologies obtained with the datasets PCG123RNA and PCG12RNA (Figure 7), because the analyses based on partitioning strategies also supported the either of 2 topologies presented (data not shown).

Phylogenetic analyses yielded unequivocal tree topology for the position of Dermaptera in Polyneoptera (Figure 7), placing as the sister to Plecoptera. This group was located either as the sister to Ephemeroptera (Figure 7A) or as the most basal lineage of the remaining polyneopteran orders (Figure 7B). The nodal support for sister group Dermaptera and Plecoptera was 0.93 ∼ 1 by BI and 85% ∼ 87% by ML analyses (Figure 7). This result is consistent with the previous findings from other molecular data. For example, Kjer [24] reported a sister relationship between Dermaptera and Plecoptera, with this group identified as the basal lineage of Neoptera (Polyneoptera, Paraneoptera, and Holometabola) on the basis of 18S rDNA. The same result also was obtained in another study using data obtained from 2 nuclear and 2 mitochondrial rRNAs, and amino acid sequences from 4 PCGs by the weighted parsimony method [27]. Another study that included newly sequenced 18S rDNA of Zoraptera also showed a sister relationship between Dermaptera and Plecoptera, placing them as the basal lineage of Neoptera [26]. However, on the basis of comprehensive morphological characteristics, Dermaptera was found to be closely related to Dictyoptera and Grylloblattodea, instead of Plecoptera [20]–[23]. Nevertheless, when the wing base structure data were analyzed independently, the sister relationship between Dermaptera and Plecoptera was supported by 2 non-homoplasious apomorphies, such as ventral basisubcostale and articulation between the antemedian total wing process and the first axillary sclerite [23]. In addition to the wing base structure, the possible synapomorphies supporting Dermaptera and Plecoptera as sister groups include 3 segmented tarsi, lack of male gonostyli and a functional ovipositor, and paired male gonopores [53].

Regarding the phylogenetic relationships among polyneopteran orders and palaeopteran orders, Odonata was always placed as the basal lineage of the Pterygota (Figure 7). This position of Odonata was also supported in recent mitochondrial genome-based phylogenetic studies on 13 basal hexapod orders and 9 basal pterygotan orders [54], [55], as well as several molecular and/or morphological studies [24], [26], [27], [56]. On the contrary, the position of Ephemeroptera was variable (Figure 7). Both BI and ML analyses based on PCG12RNA and ML analysis based on PCG123RNA supported the sister relationships between Ephmeroptera and the Polyneoptera (Figure 7B), whereas the BI analysis of PCG123RNA instead supported the sister relationships between Ephmeroptera and the group composed of (Dermaptera + Plecoptera), placed as the basal lineage of Polyneoptera (Figure 7A). Several previous studies using molecular markers and/or morphological characters have shown that Ephemeroptera is the sister to Polyneoptera, placing Odonata the sister to this group [24], [26], [27], [56]. This relationship was also supported by synapomorphic character, direct sperm transfer, shared by Ephemeroptera and Polyneoptera, while Odonata and primitive wingless hexapods possessed symplesiomorphic character, indirect sperm transfer mechanism [21]. However, recent studies using mitochondrial genomes by 1^st^ +2^nd^ codon position [54] and amino acid sequences of 13 PCGs [54], [55] without Dermaptera supported (Ephemeroptera + Plecoptera), similar to the results obtained by some of our data. With regard to the phylogenetic relationships among polyneopteran orders, the relationships within Dictyoptera (Isoptera, Mantodea, and Blattodea) were variable depending on datasets (Figure 7). Both BI and ML analyses based on dataset PCG12RNA and the ML analysis based on dataset PCG123RNA supported (Mantodea + (Isoptera + Blattodea)) (Figure 7B), whereas the BI analysis of PCG123RNA instead supported (Isoptera + (Mantodea + Blattodea)) (Figure 7A). The nodal support for both of the 2 relationships was similar (Figure 7). Both relationships have been proposed in previous studies using morphological and/or molecular data [20], [21], [26]–[28].

To clarify the 2 topologies presenting the conflicting relationships among polyneopteran and palaeopteran orders and among dictyopteran orders, topology tests [57] were performed with the 2 representative datasets, applying the GTR [58] + I + G model. The 6 statistical tests performed (ELW, BP, KH, SH, WSH, and AU) for topological characterization demonstrated that both topologies had confidence values well within the range of the 95%, suggesting that both topologies have an equal possibility, although the PCG12RNA dataset, which supports ((Ephemeroptera + Polyneoptera) + Odonata) and ((Isoptera + Blattodea) + Mantodea) (Figure 7B) obtained somewhat higher statistical confidence levels in ELW, BP, and AU tests (Table S4). Thus, a decisive conclusion on their phylogenetic relationships should be postponed until additional information from polyneopteran orders and species become available.

Except for those conflicts, the interordinal relationships within Polyneoptera were identical in all analyses (Figure 7). Dictyoptera (Isoptera + Blattodea + Mantodea), the most widely accepted superordinal group within Polyneoptera (e.g., [20], [21], [23], [28], [53], [59]), was strongly supported (1 by BI and 100% by ML analyses) as being monophyletic across all analyses. Also, the group consisting of ((Mantophasmatodea + Phasmatodea) + Grylloblattodea) was strongly supported as being monophyletic by all analyses (0.98 ∼ 1 by BI and 88% ∼ 89% by ML analyses), as first proposed by Cameron et al. [33], Kjer et al. [27], Ma et al. [35], and Gullan and Cranston [15]. This group was placed as sister to Dictyoptera in all analyses (Figure 7), which is consistent with Kjer et al. [27] and Ma et al. [35]. Nevertheless, several other studies placed either Zoraptera, Dermaptera, or Grylloblattodea together with Mantophasmatodea as the sister group to Dictyoptera using morphological characteristics and/or molecular markers [20], [21], [25], [26].

Orthoptera was supported as the sister to the group composed of (((Mantophasmatodea + Phasmatodea) + Grylloblattodea) + Dictyoptera) in all analyses with high nodal supports (1 by BI and 97% ∼ 98% by ML analyses) (Figure 7). Previous phylogenetic studies proposed a somewhat widely accepted sister relationship between Orthoptera and Phasmatodea [21]–[23], [27], [28], [59]. However, Kjer et al. [27] have shown that Orthoptera clustered together with the remaining Neoptera, excluding the very weakly supported (Dermaptera + Plecoptera) by the weighted parsimony method. This result is similar to ours in that Ortheroptera was placed as the sister to the remaining Polyenoptera, excluding Dermaptera plus Plecoptera.

## Materials and Methods

### Ethics Statement

No experiments involving vertebrate samples were performed in this study. An ethics statement is not required for the experiment which only involves an insect. The collection of the free-living earwig was permitted by the Han River Basin Environmental Office, Republic of Korea (permission number 2008–04).

### Specimens and DNA Extraction

An adult *C. fletcheri* was collected from Bukhan Mountain, Seoul, Republic of Korea in June, 2008. All necessary permits were obtained for the described field studies. DNA was extracted from a hind leg with a Wizard™ Genomic DNA Purification Kit in accordance with the manufacturer’s instructions (Promega, Madison, WI, USA).

### Generation of Sequence Data

The complete mitochondrial genome of *C. fletcheri* was sequenced with each 2 short and 2 long overlapping fragments. Each 400–600 bp of *C. fletcheri cox3* and *rrnS* (SF1 and SF2 in Figure 1, respectively) was sequenced using the primers designed from the alignment of several full-length mtDNA sequences of polyneopteran insects. The primer sequences are listed in Table 3. A polymerase chain reaction (PCR) was conducted under the following conditions: denaturation at 94°C for 7 min; 35 cycles of 94°C for 1 min, 55°C for 1 min, and 72°C for 1 min; and a final extension at 72°C for of 7 min. Based on the sequence information of SF1 and SF2, two pairs of primers were designed for the amplification of 2 overlapping long fragments, LF1 and LF2 (Figure 1; Table 3). Long PCR was performed using LA Taq™ from Takara Biomedical (Otsu, Shiga, Japan) under the following conditions: 94°C for 1 min; 30 cycles of 94°C for 30 sec and 58°C for 15 min; and a final extension step at 72°C for 12 min. The 2 long PCR fragments were then employed in the construction of a shotgun library after purification using the QIAquick PCR Purification Kit reagents (Qiagen, Germantown, MD, USA). DNA sequencing was conducted using the ABI PRISM® BigDye® Terminator ver. 3.1 Cycle Sequencing Kit and the ABI PRISM™ 3100 Genetic Analyzer from PE Applied Biosystems (Carlsbad, CA,USA). All fragments were sequenced from both strands.

**Table 3:** List of primers used to amplify long and short fragments of *C. fletcheri* mitochondrial genome.

Fragment	Primer name	Direction	Primer sequence (5′ to 3′)	Position*
SF1	Der-CO3F1	F	CWATAATYCAATGATGACG	7778 ∼ 7796
	Der-CO3R1	R	GTCCATGRAATCCTGTTGCTA	8216 ∼ 8236
SF2	Der-srRNAF1	F	CTACTWTGTACGACTTATCTC	17892 ∼ 17913
	Der-srRNAR1	R	TAAACTAGGATTAGATACCCC	18297 ∼ 18317
LF1	CF-CO3F2	F	GCATCAGGGGTTACAGTTACTTG	8035 ∼ 8057
	CF-srRNAR1	R	GAAAATGAATTCTCGACGAATAC	18009 ∼ 18031
LF2	CF-srRNAF3	F	AATCTTAAGACGACGGTATACAAGC	18134 ∼ 18158
	CF-CO3R2	R	CACAAACAGGAGAAAGACTTC	7931 ∼ 7951

* Position of primer sequences corresponds to *C. fletcheri* mitogenome; F, forward; and R, reverse.

### Genome Annotation

Sequenced fragments were assembled into a single contig with overlapping ends using the Phred+Phrap+Consed package [60]. Raw sequence data obtained were manually proof checked for indels or ambiguous base calls. The boundary of each of 13 PCGs, 2 rRNA genes, and the A+T-rich region of *C. fletcheri* was delimitated by the alignment with homologous regions of polyneopteran mitochondrial genome sequences using CLUSTAL X [61]. The nucleotide sequences of 13 PCGs were translated into amino acid sequences based on the invertebrate mtDNA genetic code using the Transseq program available at the EBI web site (http://www.ebi.ac.uk/Tools/emboss/transeq/). Delimitation and prediction of secondary structure of tRNAs, except for *trnS*(AGN), were performed by tRNAscan-SE 1.21 using invertebrate mitochondrial codon predictors and a cove score cut off of 1 [62]. The *trnS*(AGN) was determined via sequence comparison with published polyneopteran mitochondrial genomes, and its secondary structure was predicted manually. The sequence data is deposited in the GenBank database (GenBank: JN651407).

### Comparative Sequence Analysis

The A/T-content in 13 PCGs, 2 rRNAs, the A+T-rich region, and whole genome, were calculated using the EditSeq program in the Lasergene software package (www.dnastar.com). The compositional skew for whole PCGs, and major-strand and minor-strand encoded PCGs was calculated with the following formula: GC-skewness  =  (G - C)/(G + C) and AT-skewness  =  (A - T)/(A + T), where C, G, T and A are the frequencies of each nucleotide [63]. The nucleotide composition at each codon position of the PCGs was calculated using PAUP ver. 4.0b10 [64]. The overlapping regions and intergenic spacers between genes were counted manually. Tandem repeat units in the genome were searched using Tandem Repeats Finder program [65]. The stem-and-loop structures in the A+T-rich region were predicted by the web server Mfold [66].

### Phylogenetic Analysis

A total of 13 polyneopteran mitochondrial genomes representing 9 orders were employed to reconstruct the phylogenetic relationships of Polyneoptera using BI and ML algorithms. The mitochondrial genome of embiopteran species, *Aposthonia japonica* [14] was not included in the analyses because it just became available during final phase of our manuscript. With the consideration that the Ephemeroptera has been proposed to be the sister to Plecoptera [54], [55] under the controversial relationships among Neoptera, Ephemeroptera, and Odonata [21], [27], [28] and that the monophyly of Ectognatha (Pterygota, Archaeognatha, and Zygentoma) is widely accepted [67], the two palaeopteran orders Odonata and Ephemeroptera, and each 2 species of Archaeognatha [68], [69] and Zygentoma [70], [71] were also included as ingroup taxa in the analyses. 2 species of Diplura [72], [73] and 3 species of Collembola [74]–[76], which have been used to root pterygotan phylogeny [54] were utilized as outgroups,. Considering the previous suggestions that either all mitochondrial genome data (all PCGs, rRNAs, and tRNAs) or 1^st^ +2^nd^ codon positions of 13 PCGs are better utilized to infer ordinal relationships of holometabolous orders or basal pterygotan orders [54], [77], the datasets PCG123RNA (all codon positions of 13 PCGs plus all rRNAs and tRNAs) and PCG12RNA (1^st^ +2^nd^ codon positions plus all rRNAs and tRNAs) were generated for phylogenetic analyses for Polyneoptera and Palaeoptera. Both datasets were divided into either 2 partitions dividing 13 PCGs into one and RNAs into another partition or 14 partitions dividing each PCGs and one for RNAs for BI analyses.

To obtain codon-based alignment for each PCG, the nucleotide sequence of each PCG was subjected to RevTrans ver. 1.4 [78]. The well-aligned conserved blocks of each PCG were selected using GBlocks 0.91b [79]. Substitution model selection was conducted via comparison of Akalike Information Criterion (AIC) scores [80] using Modeltest ver. 3.7 [81]. For datasets PCG123RNA and PCG12RNA, the GTR [58] + I + G was selected as the best-fit model for both ML and BI analyses. The ML and BI analyses were conducted using RAxML [82] and MrBayes ver. 3.1 [83], respectively, as previously described [54], [84]. When the partition option was employed, each partition was unlinked with the model selected by ModelTest [81] applied (data not shown).

Because discordant topologies were obtained depending on datasets, those topologies were subjected to topology tests using the ML method incorporated in Treefinder [57], applying the GTR [58] + I + G model. The statistical confidence values of each topology were determined via 6 statistical tests, each with 1,000 replications: ELW [85], BP [86], KH [87], SH [88], WSH [88], and AU [89].

## Supporting Information

Figure S1 **Stem-and-loop structures found in the A+T-rich region of** ***C. fletcheri*** **mitochondrial genome.** The underline and dashed lines indicate the identical and similar flanking sequences in the stem-and-loop structures, respectively, that have conservatively been found in Orthoptera and Diptera. The numbers in parenthesis indicate the number of redundant structures. The nucleotide position is indicated at the beginning and end sites of the structures.
(PDF)

Figure S2 **Mitochondrial gene arrangement in Polyneoptera.** Gene sizes are not drawn to scale. tRNA genes are abbreviated using the one-letter amino acid code, with L  =  *trnL*(CUN); L*  =  *trnL*(UUR); S  =  *trnS*(AGN); S*  =  *trnS*(UCN). Gene names that are not underlined indicate a forward direction, whereas underlines indicate a reverse transcriptional direction. CR indicates the A+T-rich region.
(PDF)

Table S1 **Nucleotide composition and skewness of mitochondrial genomes of Polyneoptera.**
(PDF)

Table S2 **Nucleotide composition at each codon position of the concatenated 13 PCGs in Polyneoptera.**
(PDF)

Table S3 **Composition and skewness of mitochondrial PCGs in Polyneoptera.**
(PDF)

Table S4 **Results of topological tests for 2 datasets, showing values from 6 statistical tests performed.**
(PDF)
