# Supplementary figures and images for: Complete Mitochondrial Genome of the Free-Living Earwig, Challia fletcheri (Dermaptera: Pygidicranidae) and Phylogeny of Polyneoptera
Source: PLoS One. 2012 Aug 6;7(8):e42056. doi: 10.1371/journal.pone.0042056 (PMC3412835; doi:10.1371/journal.pone.0042056)

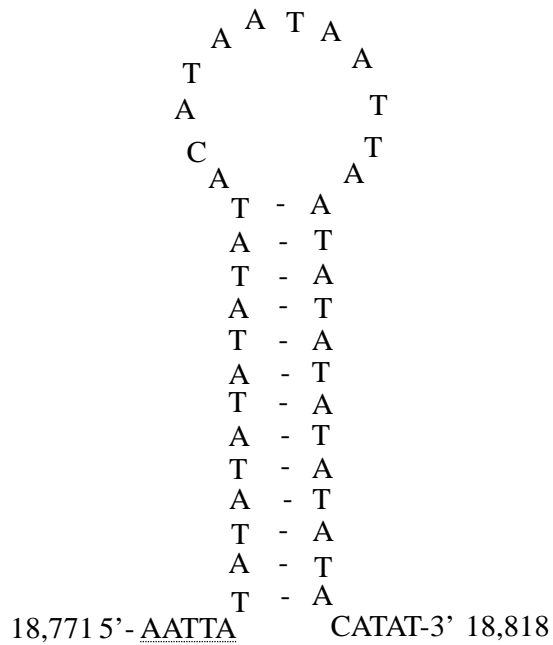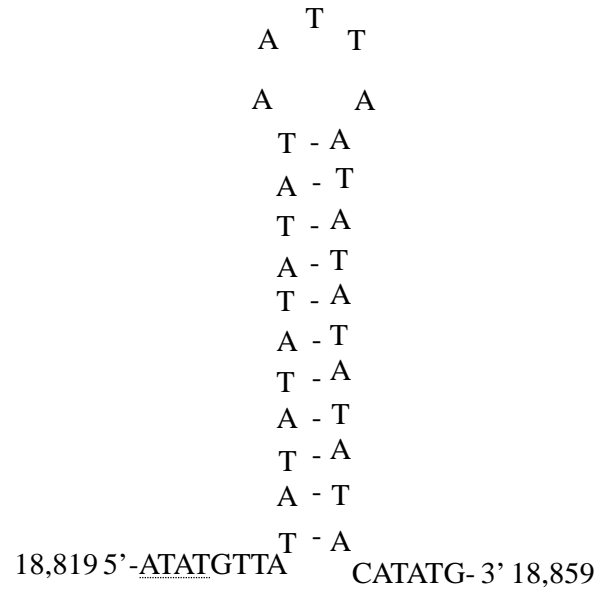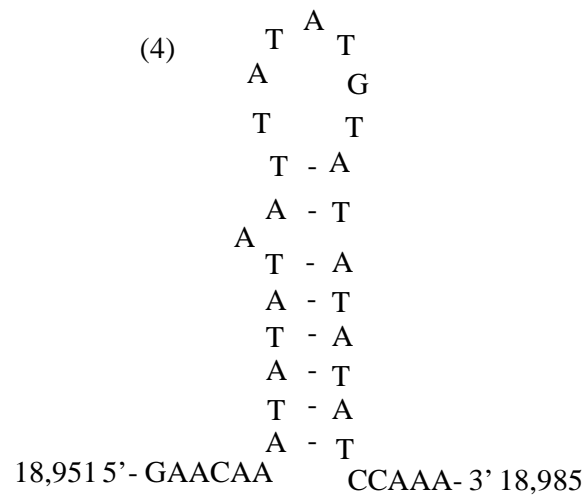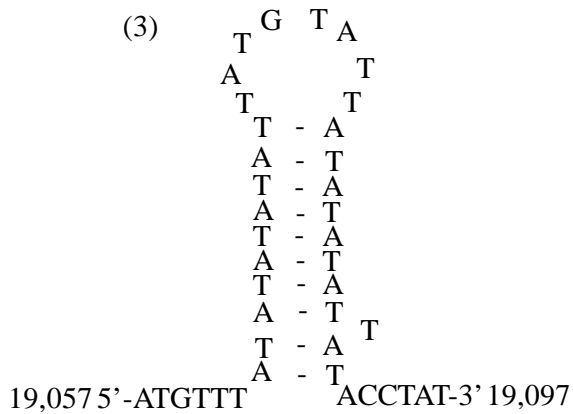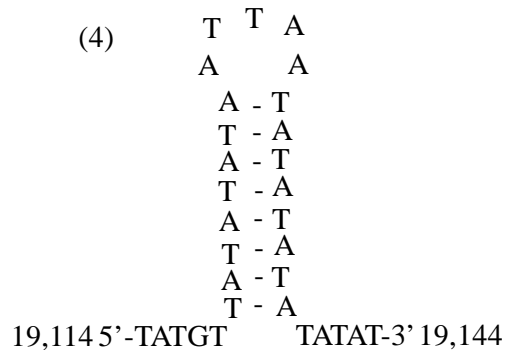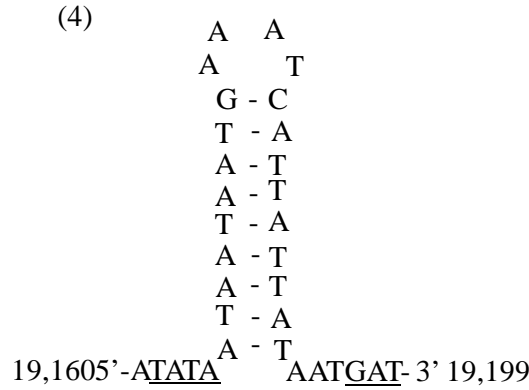

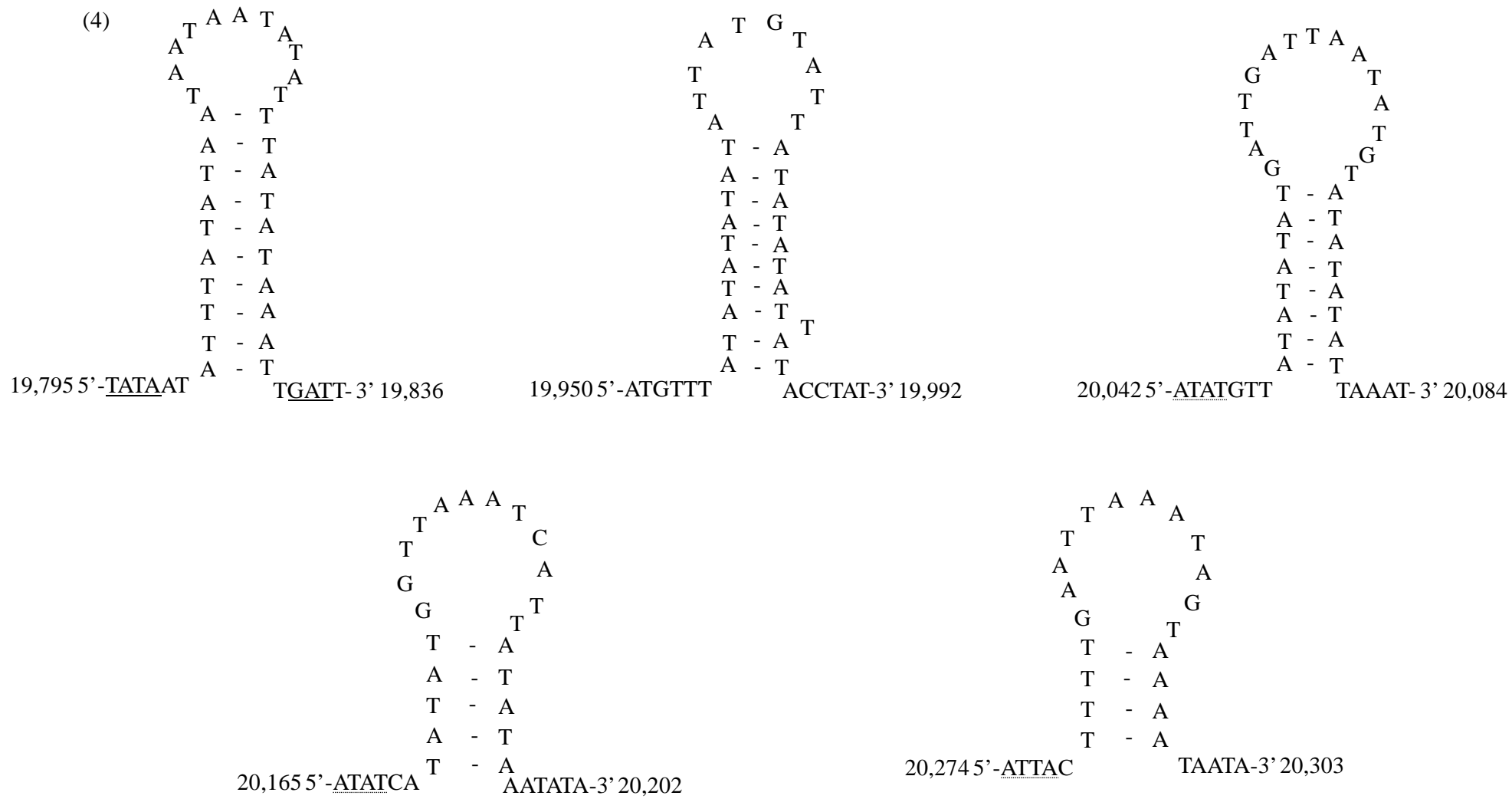

Figure S1

Supplement: Figure S1 — Stem-and-loop structures found in the A+T-rich region of C. fletcheri mitochondrial genome. The underline and dashed lines indicate the identical and similar flanking sequences in the stem-and-loop structures, respectively, that have conservatively been found in Orthoptera and Diptera. The numbers in parenthesis indicate the number of redundant structures. The nucleotide position is indicated at the beginning and end sites of the structures. (PDF) [file pone.0042056.s001.pdf]
