# Supplementary material for: Complete Mitochondrial Genome of the Free-Living Earwig, Challia fletcheri (Dermaptera: Pygidicranidae) and Phylogeny of Polyneoptera
Source: PLoS One. 2012 Aug 6;7(8):e42056. doi: 10.1371/journal.pone.0042056 (PMC3412835; doi:10.1371/journal.pone.0042056)
Supplement: Figure S2 — Mitochondrial gene arrangement in Polyneoptera. Gene sizes are not drawn to scale. tRNA genes are abbreviated using the one-letter amino acid code, with L = trnL(CUN); L* = trnL(UUR); S = trnS(AGN); S* = trnS(UCN). Gene names that are not underlined indicate a forward direction, whereas underlines indicate a reverse transcriptional direction. CR indicates the A+T-rich region. (PDF) [file pone.0042056.s002.pdf]

Ancestral arrangement

I	Q	M	*nad2*	W	C	Y	*cox1*	L*	*cox2*	K	D	*atp8*	*atp6*	*cox3*	G	*nad3*	A	R	N	S	E	F	*nad5*	H	*nad4*	*nad4L*	T	P	*nad6*	*cytb*	S*	*nad1*	L	*rrnL*	V	*rrnS*	CR

Orthoptera: Ensifera

I	Q	M	*nad2*	W	C	Y	*cox1*	L*	*cox2*	K	D	*atp8*	*atp6*	*cox3*	G	*nad3*	A	R	E	S	N	F	*nad5*	H	*nad4*	*nad4L*	T	P	*nad6*	*cytb*	S*	*nad1*	L	*rrnL*	V	*rrnS*	CR

Teleogryllus emma

Orthoptera: Caelifera (Acridomorpha)

I	Q	M	*nad2*	W	C	Y	*cox1*	L*	*cox2*	D	K	*atp8*	*atp6*	*cox3*	G	*nad3*	A	R	N	S	E	F	*nad5*	H	*nad4*	*nad4L*	T	P	*nad6*	*cytb*	S*	*nad1*	L	*rrnL*	V	*rrnS*	CR

Oxya chinensis; *Locusta migratoria*; *Calliptamus italicus*; *Acrida willemsei*; *Phlaeoba albonema*; *Locusta migratoria migratoria*; *Schistocerca gregaria*; *Gomphocerus licenti*; *Prumna arctica*; *Traulia szetschuanensis*; *Arcyptera coreana*; *Gastrimargus marmoratus*; *Oedaleus decorus asi aticus*; *Ognevia longipennis*; *Atractomorpha sinensis*; *Chorthippus chinensis*; *Oxya chinensis*

Figure S2
