# Supplementary material for: Complete Mitochondrial Genome of the Free-Living Earwig, Challia fletcheri (Dermaptera: Pygidicranidae) and Phylogeny of Polyneoptera
Source: PLoS One. 2012 Aug 6;7(8):e42056. doi: 10.1371/journal.pone.0042056 (PMC3412835; doi:10.1371/journal.pone.0042056)
Supplement: Table S1 — Nucleotide composition and skewness of mitochondrial genomes of Polyneoptera. (PDF) [file pone.0042056.s003.pdf]

Table S1 Nucleotide composition and skewness of mitochondrial genomes of Polyneoptera.

Taxon	Whole genome				PCGs		*rrnL*		*rrnS*		A+T-rich region		Accession number	References
	Size (bp)	AT%	AT-skewness	GC-skewness	No. codons[a]	AT%	Size (bp)	AT%	Size (bp)	AT%	Size (bp)	AT%		
Polyneoptera														
**Dermaptera**														
Challia fletcheri	**20,456**	**72.5**	**-0.166**	**0.346**	**3,728**	**68.5**	**1,334**	**70.9**	**763**	**70.2**	**1,816**	**89.3**	**JN651407**	**This study**
Plecoptera														
Pteronarcys princeps	16,004	71.5	0.038	-0.224	3,729	70.4	1,328	73.9	793	68.6	1,158	81.3	AY687866	[1]
Mantophasmatodea														
Sclerophasma paresisense	15,500	75.1	0.108	-0.152	3,709	74.0	1,313,	77.2	781	75.3	715	84.1	DQ241798	[2]
Mantodea														
Tamolanica tamolana	16,055	75.3	0.059	-0.236	3,706	74.7	1,318	77.8	777	75.7	970	74.7	DQ241797	[2]
Blattodea														
Periplaneta fuliginosa	14,996	75.2	0.121	-0.167	3,712	74.5	1,300	78.5	801	75.8	208*	76.9	AB126004	[3]
Blattella germanica	15,025	74.6	0.051	-0.180	3,703	74.2	1,300	78.2	794	73.2	253	75.1	EU854321	Unpublished
Eupolyphaga sinensis	15,553	72.0	0.122	-0.250	3,721	70.9	1,293	74.7	801	72.9	857	77.8	FJ830540	[4]
Grylloblattodea														
Grylloblatta sculleni	15,595	70.1	0.104	-0.194	3,715	69.1	1,313	69.7	742	66.4	956*	67.0	DQ241796	[2]
Phasmatodea														
Timema californicum	14,387	72.1	0.078	-0.179	3,721	70.7	1,318	77.8	472*	71.8	-	-	DQ241799	[2]
Ramulus hainanense	15,590	73.1	0.176	-0.215	3,685	71.6	1,287	78.4	782	76.6	977	74.7	FJ156750	Unpublished
Phraortes sp. Iriomote	16,867	78.8	0.182	-0.142	3,677	77.2	1,275	80.3	759	79.1	2352	84.4	AB477464	[5]
Ramulus irregulariterdentatus	16,633	76.0	0.136	-0.110	3,694	75.5	1,291	79.8	781	76.7	2,026	74.0	AB477463	[5]
Phraortes illepidus	16,456	77.6	0.178	-0.167	3,681	76.6	1,279	79.9	764	76.1	1,903	81.7	AB477460	[5]
Entoria okinawaensis	16,910	76.0	0.154	-0.152	3,692	75.6	1,286	79.4	780	77.7	2,304	73.1	AB477459	[5]
Megacrania alpheus adan	17,124	76.9	0.203	-0.198	3,695	75.5	1,288	79.3	777	76.5	2,520	80.6	AB477471	[5]
Heteropteryx dilatata	16,618	76.1	0.186	-0.205	3,691	75.6	1,305	78.2	776	75.0	1,974	76.7	AB477468	[5]
Phobaeticus serratipes	16,182	77.0	0.192	-0.219	3,681	75.9	1,286	80.2	758	77.6	1,608	80.7	AB477467	[5]
Micadina phluctainoides	16,507	78.5	0.165	-0.098	3,692	76.8	1,284	81.2	764	78.8	1,842	85.0	AB477466	[5]
Isoptera														
Reticulitermes santonensis	16,567	66.1	0.303	-0.293	3,711	64.5	1,313	69.4	743	66.5	1,761	70.9	EF206315	[6]
Reticulitermes hageni	16,590	65.5	0.300	-0.287	3,711	63.7	1,315	69.9	748	67.0	1,773	70.1	EF206320	[6]
Reticulitermes virginicus	16,573	65.9	0.298	-0.295	3,711	64.7	1,309	69.3	745	66.0	1,700	68.4	EF206318	[6]
Reticulitermes flavipes	16,565	66.2	0.300	-0.292	3,711	64.7	1,313	69.7	742	66.4	1,760	70.5	EF206314	[6]
Orthoptera														
Ensifera														
Deracantha onos	15,650	69.2	0.036	-0.287	3,729	67.7	1,301	72.3	858	70.1	815	77.8	EU137664	[7]
Troglophilus neglectus	15,810	73.4	0.011	-0.260	3,727	72.5	1,342	76.8	785	73.1	539	66.8	EU938374	[8]
Anabrus simplex	15,766	69.4	0.030	-0.276	3,725	67.6	1,312	72.9	785	68.9	986	80.1	EF373911	[9]
Gampsocleis gratiosa	15,929	65.3	0.062	-0.300	3,729	63.6	1,317	70.0	848	67.7	1,111	67.4	EU527333	[10]
Ruspolia dubia	14,971	70.9	0.023	-0.240	3,728	69.9	1,302	74.4	881	72.2	70	71.4	EF583824	[11]

Myrmecophilus manni	15,323	70.2	0.068	-0.290	3,677	69.0	1,252	74.1	734	69.8	789	74.5	EU938370	[8]
Gryllotalpa orientalis	15,521	70.5	0.045	-0.302	3,672	69.4	1,247	72.5	719	69.4	920	74.9	AY660929	[12]
Teleogryllus emma	15,660	73.1	0.108	-0.268	3,701	72.6	1,293	75.3	812	71.7	997	74.0	EU557269	Unpublished
Gryllotalpa pluvialis	15,525	72.2	0.042	-0.306	3,689	71.1	1,236	74.9	783	72.0	866	77.7	EU938371	[8]
Elimaea cheni	15,831	72.6	0.021	-0.260	3,731	71.5	1,311	76.3	828	74.5	999	75.5	GU323362	[13]
Caelifera														
Acrida willemsei	15,601	76.2	0.165	-0.163	3,719	75.4	1,314	78.1	718	74.9	848	87.3	EU938372	[8]
Schistocerca gregaria gregaria	15,625	73.2	0.155	-0.167	3,718	72.2	1,320	75.8	813	71.1	762	87.0	GQ491031	[14]
Gomphocerus licenti	15,597	74.8	0.141	-0.143	3,716	74.3	1,315	77.4	847	73.4	712	83.7	GQ180102	Unpublished
Arcyptera coreana	15,783	76.3	0.126	-0.133	3,719	75.8	1,309	77.9	792	74.0	945	85.7	GU324311	Unpublished
Chorthippus chinensis	15,599	75.1	0.141	-0.142	3,675	74.9	1,313	77.0	843	74.7	721	84.1	EU029161	Unpublished
Phlaeoba albonema	15,657	74.1	0.150	-0.165	3,721	73.5	1,312	76.1	844	13.8	728	83.0	EU370925	Unpublished
Oxya chinensis	15,443	75.9	0.123	-0.132	3,728	75.1	1,317	78.6	778	75.3	631	87.5	EF437157	[15]
Calliptamus italicus	15,675	73.2	0.139	-0.162	3,717	72.4	1,322	75.6	801	70.5	782	85.8	EU938373	[8]
Primnoa arctica	15,628	76.1	0.134	-0.139	3,719	75.2	1,312	78.8	848	75.6	744	88.2	GU294758	[16]
Traulia szetschuanensis	15,768	74.6	0.143	-0.164	3,715	73.9	1,314	77.3	814	72.5	922	82.5	EU914849	Unpublished
Ognevia longipennis	15,621	76.0	0.118	-0.145	3,711	75.3	1,313	77.1	820	75.5	774	87.6	EU914848	Unpublished
Oedaleus asiaticus	16,259	75.4	0.195	-0.184	3,716	73.9	1,318	78.2	831	75.5	1,401	84.5	EU513374	[17]
Gastrimargus marmoratus	15,924	75.2	0.212	-0.226	3,725	73.9	1,322	78.1	831	75.7	1,061	84.3	EU513373	[17]
Locusta migratoria migratoria	16,053	75.5	0.186	-0.190	3,713	74.2	1,316	78.5	834	76.3	1243	84.6	EU287446	Unpublished
Locusta migratoria	15,722	75.3	0.183	-0.182	3,713	74.1	1,314	74.1	982	78.8	926	85.9	X80245	[18]
Locusta migratoria tibetensis	15,568	75.4	0.176	-0.175	3,715	74.2	1,316	78.5	833	76.0	711	86.4	HM219224	Unpublished
Locusta migratoria manilensis	15,895	75.3	0.186	-0.185	3,729	74.1	1,318	78.7	831	75.5	1,034	84.8	GU344101	Unpublished
Atractomorpha sinensis	15,558	74.3	0.161	-0.175	3,712	73.6	1,311	76.2	819	74.9	777	81.5	EU263919	Unpublished
Gomphocerus sibiricus	15,571	74.7	0.137	-0.136	3,720	74.3	1,315	77.4	823	73.0	706	82.3	HM131804	Unpublished
Acrida cinerea	15,599	76.1	0.166	-0.159	3,720	75.3	1,316	76.8	782	75.5	784	87.9	GU344100	[19]
Thrinchus schrenkii	15,672	71.3	0.166	-0.205	3,721	70.7	1,319	72.7	852	70.7	748	78.6	GU181288	[20]
Physemacris variolosa	17,004	75.5	0.158	-0.245	3,711	73.3	1,313	76.7	749	74.8	2,277	85.4	GU945504	[21]
Xyleus modestus	15,723	72.4	0.171	-0.193	3,714	71.3	1,326	74.5	805	70.4	621	84.7	GU945503	[21]
Mekongiella xizangensis	15,885	73.6	0.157	-0.212	3,704	72.8	1,327	75.7	834	73.3	1,063	78.7	HM583654	[22]
Mekongiana xiangchengensis	15,567	74.6	0.146	-0.187	3,717	73.9	1,331	77.2	828	73.8	733	82.8	HM583653	[22]
Euchorthippus fusigeniculatus	15,772	75.0	0.137	-0.151	3,713	74.4	1,315	77.7	832	74.8	893	81.6	HM583652	[22]
Gomphocerippus rufus	15,598	74.4	0.141	-0.156	3,721	73.6	1,311	77.4	846	73.8	695	82.7	GU294759	[16]
Ellipes minuta	15,451	66.5	0.012	-0.297	3,722	64.9	1,304	70.8	777	70.1	582	71.1	GU945502	[21]

[a]Termination codons were excluded in total codon count; [b]Protein coding genes; -, A+T-rich region was not sequenced or defined; *, partial sequences.

References

1. Stewart JB, Beckenbach AT (2006) Insect mitochondrial genomics 2: the complete mitochondrial genome sequence of a giant stonefly, *Pteronarcys princeps*, asymmetric directional mutation bias, and conserved plecopteran A+T-region elements. Genome 49: 815-824.

2. Cameron SL, Barker SC, Whiting MF (2006) Mitochondrial genomics and the new insect order Mantophasmatodea. Mol Phylogenet Evol 38: 274-279.

3. Yamauchi MM, Miya MU, Nishida M (2004) Use of a PCR-based approach for sequencing whole mitochondrial of insects: two examples (cockroach and dragonfly) based on method developed for decapod crustaceans. Insect Mol Biol 13: 435-442.

4. Zhang YY, Xuan WJ, Zhao JL, Zhu CD, Jiang GF (2010) The complete mitochondrial genome of the cockroach *Eupolyphaga sinensis* (Blattaria: Polyphagidae) and the phylogenetic relationships within the Dictyoptera. Mol Biol Rep 37: 3509-3516.

5. Kômoto N, Yukuhiro K, Ueda K, Tomita S (2010) Exploring the molecular phylogeny of phasmids with whole mitochondrial genome sequences. Mol Phylogenet Evol 58: 43-52.

6. Cameron SL, Whiting MF (2007) Mitochondrial genomic comparisons of the subterranean termites from the genus *Reticulitermes* (Insecta: Isoptera: Rhinotermitidae). Genome 50: 188-202.

7. Zhou Z, Huang Y, Shi F, Ye H (2009) The complete mitochondrial genome of *Deracantha onos* (Orthoptera: Bradyporidae). Mol Biol Rep 36: 7-12.

8. Fenn JD, Song H, Cameron SL, Whiting MF (2008) A preliminary mitochondrial genome phylogeny of Orthoptera (Insecta) and approaches to maximizing phylogenetic signal found within mitochondrial genome data. Mol Phylogenet Evol 49: 59-68.

9. Cameron SL, Barker SC, Whiting MF (2006) Mitochondrial genomics and the new insect order Mantophasmatodea. Mol Phylogenet Evol 38: 274-279.

10. Zhou Z, Shi F, Huang Y (2008) The complete mitogenome of the Chinese bush cricket, *Gampsocleis gratiosa* (Orthoptera: Tettigonioidea). J Genet Genomics 35: 341-348.

11. Zhou Z, Huang Y, Shi F (2007) The mitochondrial genome of *Ruspolia dubia* (Orthoptera: Conocephalidae) contains a short A+T-rich region of 70 bp in length. Genome 50: 855-66.

12. Kim I, Cha SY, Yoon MH, Hwang JS, Lee SM, et al. (2005) The complete nucleotide sequence and gene organization of the mitochondrial genome of the oriental mole cricket, *Gryllotalpa orientalis* (Orthoptera: Gryllotalpidea). Gene 353: 155-168.

13. Zhou Z, Ye H, Huang Y, Shi F (2010) The phylogeny of Orthoptera inferred from mtDNA and description of Elimaea cheni (Tettigoniidae: Phaneropterinae) mitogenome. J Genet Genomics 37: 315-324.

14. Erler S, Ferenz HJ, Moritz RFA, Kaatz HH (2010) Analysis of the mitochondrial genome of *Schistocerca gregaria gregaria* (Orthoptera: Acrididae). Biol J Linn Soc Lond 99: 296-305.

15. Zhang C, Huang Y (2008) Complete mitochondrial genome of *Oxya chinensis* (Orthoptera, Acridoidea). Acta Biochim Biophys Sin 40: 7-18.

16. Sun H, Zheng Z, Huang Y (2010) Sequence and phylogenetic analysis of complete mitochondrial DNA genomes of two grasshopper species *Gomphocerus rufus* (Linnaeus, 1758) and *Primnoa arctica* (Zhang and Jin, 1985) (Orthoptera: Acridoidea). Mitochondr DNA 21: 115-31.

17. Ma C, Liu C, Yang P, Kang L (2009) The complete mitochondrial genomes of two band-winged grasshoppers, *Gastrimargus marmoratus* and *Oedaleus asiaticus*. BMC Genomics 10: 156.

18. Flook PK, Klee S, Rowell CHF (1999) Combined molecular phylogenetic analysis of the Orthoptera and its implications for their higher systematics. Syst Biol 48: 233-253.

19. Liu N, Huang, Y (2010) Complete mitochondrial genome sequence of *Acrida cinerea* (Acrididae: Orthoptera) and comparative analysis of mitochondrial genomes in Orthoptera. Comp Funct Genomics 2010: 319486.

20. Zhang D, Zhi Y, Yin H, Li X, Yin X (2011) The complete mitochondrial genome of *Thrinchus schrenkii* (Orthoptera: Caelifera, Acridoidea,

Pamphagidae). Mol Biol Rep 38: 611-619.

21. Sheffield NC, Hiatt KD, Valentine MC, Song H, Whiting MF (2010) Mitochondrial genomics in Orthoptera using MOSAS. Mitochondr DNA 21: 87-104.

22. Zhao L, Zheng ZM, HuangY, Sun HM (2010) A comparative analysis of mitochondrial genomes in Orthoptera (Arthropoda: lnsecta) and genome descriptions of three Grasshopper species. Zool Sci 27: 662-672.
