# Supplementary material for: Complete Mitochondrial Genome of the Free-Living Earwig, Challia fletcheri (Dermaptera: Pygidicranidae) and Phylogeny of Polyneoptera
Source: PLoS One. 2012 Aug 6;7(8):e42056. doi: 10.1371/journal.pone.0042056 (PMC3412835; doi:10.1371/journal.pone.0042056)
Supplement: Table S2 — Nucleotide composition at each codon position of the concatenated 13 PCGs in Polyneoptera. (PDF) [file pone.0042056.s004.pdf]

Table S2 Nucleotide composition at each codon position of the concatenated 13 PCGs in Polyneoptera.

Species	1st codon position				2nd codon position				3rd codon position				Overall			
	A	T	C	G	A	T	C	G	A	T	C	G	A	T	C	G
**Dermaptera**																
Challia fletcheri	**30.9**	**31.6**	**14.2**	**23.3**	**18.3**	**45.6**	**18.5**	**17.6**	**35.5**	**43.8**	**8.9**	**11.8**	**28.2**	**40.3**	**13.9**	**17.6**
Plecoptera																
Pteronarcys princeps	28.2	35.0	14.4	22.3	18.5	44.8	21.4	15.2	39.9	44.8	8.9	6.5	28.9	41.6	14.9	14.7
Mantophasmatodea																
Sclerophasma paresisense	32.9	35.7	12.0	19.4	19.6	46.9	19.3	14.2	43.6	43.4	7.7	5.3	32.0	42.0	13.0	13.0
Mantodea																
Tamolanica tamolana	32.2	36.5	11.6	19.7	20.2	46.3	19.0	14.4	44.1	44.8	7.4	3.7	32.2	42.5	12.7	12.6
Blattodea																
Periplaneta fuliginosa	33.0	34.5	12.3	20.2	19.7	46.3	19.1	14.9	45.7	44.4	5.6	4.4	32.8	41.8	12.3	13.2
Blattella germanica	31.6	36.3	11.9	20.3	19.4	46.0	19.6	14.9	43.5	45.8	6.4	4.4	31.5	42.7	12.7	13.2
Eupolyphaga sinensis	33.0	33.9	13.1	20.1	19.9	45.4	19.6	15.1	39.5	41.4	10.6	8.8	30.8	40.1	14.4	14.7
Grylloblattodea																
Grylloblatta sculleni	31.4	33.7	14.6	20.3	18.8	45.6	20.2	15.4	37.5	40.2	12.4	9.9	29.2	39.8	15.7	15.2
Phasmatodea																
Timema californicum	33.5	33.4	13.7	19.4	19.6	46.6	19.4	14.4	38.1	41.0	11.3	9.5	30.4	40.3	14.8	14.5
Ramulus hainanense	35.8	32.7	13.0	18.5	20.3	46.2	19.4	14.1	39.9	39.8	11.2	9.2	32.0	39.6	14.5	13.9
Phraortes sp. Iriomote	38.5	34.6	10.8	16.2	20.7	48.0	17.9	13.5	45.9	44.0	6.0	4.2	35.0	42.2	11.5	11.3
Ramulus irregulariterdentatus	37.1	34.1	11.6	17.3	20.4	47.2	18.4	13.9	43.8	43.9	6.8	5.4	33.8	41.7	12.3	12.2
Phraortes illepidus	37.1	34.3	11.5	17.1	20.9	47.5	18.0	13.6	45.3	44.6	6.0	4.1	34.4	42.1	11.8	11.6
Entoria okinawaensis	36.5	34.3	11.6	17.7	20.8	47.1	18.6	13.5	43.9	44.3	7.0	4.8	33.7	41.9	12.4	12.0
Megacrania alpheus adan	37.8	33.1	11.9	17.1	20.9	47.1	18.1	13.9	45.1	42.4	7.1	5.4	34.6	40.9	12.4	12.1
Heteropteryx dilatata	37.1	34.2	11.2	17.5	20.5	47.2	18.8	13.6	44.7	43.0	7.3	5.0	34.1	41.5	12.4	12.0
Phobaeticus serratipes	37.3	34.9	11.1	16.7	20.3	47.6	18.0	14.1	45.9	41.7	7.3	5.1	34.5	41.4	12.1	12.0
Micadina phluctainoides	36.7	35.5	11.1	16.6	20.7	47.5	13.8	18.0	46.3	43.5	5.7	4.5	34.6	42.2	11.6	11.7
Isoptera																

Reticulitermes santonensis	30.3	30.3	16.4	23.0	18.4	43.8	21.8	16.0	38.4	32.3	17.5	11.8	29.0	35.4	18.6	17.0
Reticulitermes hageni	30.1	29.9	17.0	23.1	18.4	43.7	21.9	16.1	37.7	31.5	18.0	12.8	28.7	35.0	18.9	17.3
Reticulitermes virginicus	30.4	30.2	16.7	22.8	18.4	43.8	21.8	16.0	39.0	32.2	17.2	11.6	29.2	35.4	18.5	16.8
Reticulitermes flavipes	30.4	30.2	16.5	23.0	18.4	43.8	21.8	16.1	38.8	32.3	17.4	11.4	29.2	35.4	18.5	16.8
Orthoptera																
Deracantha onos	30.2	33.2	15.5	21.2	19.2	45.2	20.7	14.9	37.3	38.3	15.2	9.2	28.9	38.9	17.1	15.1
Troglophilus neglectus	30.6	34.3	14.6	20.6	19.1	45.2	20.9	14.9	41.9	46.4	7.5	4.2	30.5	42.0	14.3	13.2
Anabrus simplex	30.0	32.7	15.7	21.6	19.0	45.4	20.9	14.7	35.1	40.6	14.7	9.6	28.1	39.6	17.1	15.3
Gampsocleis gratiosa	29.6	31.1	17.2	22.1	18.9	44.9	21.0	15.2	31.8	34.5	20.6	13.2	26.7	36.8	19.6	16.8
Ruspolia dubia	29.5	34.5	14.2	21.8	19.0	45.2	20.8	15.0	39.0	42.6	11.2	7.3	29.2	40.7	15.4	14.7
Myrmecophilus manni	32.1	32.0	15.6	20.2	18.6	46.0	20.3	15.1	38.5	39.7	13.7	8.1	29.7	39.2	16.6	14.5
Gryllotalpa orientalis	30.5	33.8	15.0	20.8	18.9	45.9	20.2	15.1	38.1	41.1	12.7	8.1	29.2	40.3	16.0	14.6
Teleogryllus emma	31.8	36.5	13.4	18.5	23.3	44.5	17.5	14.7	38.0	43.6	10.6	7.8	31.0	41.5	13.8	13.6
Gryllotalpa pluvialis	30.6	34.5	14.3	20.6	19.0	45.9	20.0	15.2	40.3	43.3	10.3	6.2	29.9	41.2	14.9	14.0
Elimaea cheni	30.6	35.3	13.8	20.4	19.2	45.7	20.0	15.1	41.1	42.5	9.7	6.7	30.3	41.2	14.5	14.1
Acrida willemsei	34.5	35.3	11.5	18.6	20.0	46.1	19.8	14.2	45.0	45.2	5.6	4.2	33.2	42.2	12.3	12.3
Schistocerca gregaria	31.3	33.8	13.7	21.2	19.6	45.2	20.8	14.5	42.3	44.2	8.0	5.4	31.1	41.1	14.2	13.7
Gomphocerus licenti	31.4	35.7	12.4	20.5	19.7	46.0	20.1	14.2	43.3	46.7	5.5	4.5	31.4	42.8	12.7	13.1
Arcyptera coreana	34.1	35.3	11.7	19.0	19.8	46.3	19.7	14.2	42.2	46.7	5.0	3.1	33.0	42.8	12.1	12.1
Chorthippus chinensis	33.9	35.8	11.8	18.5	19.8	46.2	20.3	13.8	43.4	45.9	5.6	5.1	32.3	42.6	12.6	12.5
Phlaeoba albonema	32.5	34.7	12.6	20.3	19.8	45.4	20.3	14.5	43.5	44.6	6.7	5.2	31.9	41.6	13.2	13.3
Oxya chinensis	32.3	36.4	11.6	19.8	19.7	46.1	20.0	14.3	43.9	47.2	5.2	3.7	32.0	43.2	12.2	12.6
Calliptamus italicus	31.2	34.7	13.1	21.0	19.5	45.4	20.6	14.5	42.7	43.6	8.1	5.6	31.2	41.2	13.9	13.7
Prumna arctica	33.1	35.7	12.4	18.8	19.8	46.3	19.8	14.1	44.2	46.5	5.7	3.7	32.4	42.8	12.6	12.2
Traulia szetschuanensis	32.9	35.1	12.5	19.5	19.7	46.0	20.0	14.3	43.3	44.7	6.6	5.4	32.0	41.9	13.1	13.1
Ognevia longipennis	33.3	35.9	12.1	18.7	19.8	46.8	19.2	14.3	43.7	46.2	5.5	4.5	32.3	43.0	12.3	12.5
Oedaleus decorus asiaticus	34.2	34.4	12.5	18.3	19.9	45.7	20.2	14.2	45.8	41.5	8.1	4.6	45.0	30.4	14.6	10.0
Gastrimargus marmoratus	34.5	34.8	12.3	18.4	20.2	45.7	19.9	14.2	45.2	41.3	8.8	4.7	33.3	40.6	13.7	12.4
Locusta migratoria migratoria	33.8	35.0	12.5	18.8	20.4	45.8	19.9	14.0	44.5	43.2	8.0	4.3	32.9	41.2	13.5	12.4
Locusta migratoria	33.8	35.0	12.4	18.8	20.4	45.7	20.0	19.9	44.4	43.0	8.0	4.6	32.9	41.3	13.5	12.3

Locusta migratoria tibetensis	33.7	34.9	12.5	18.9	20.4	45.8	19.8	14.0	44.7	43.1	8.1	4.2	32.9	41.3	13.5	12.4
Locusta migratoria manilensis	33.8	35.0	12.4	18.8	20.2	45.8	19.9	14.1	44.7	42.8	8.1	4.4	32.9	41.2	13.5	12.4
Atractomorpha sinensis	33.8	35.6	11.8	18.8	19.8	46.4	19.3	14.5	41.5	43.5	8.4	6.6	31.7	41.9	13.2	13.3
Gomphocerus sibiricus	32.6	35.2	12.3	20.0	19.7	45.8	20.3	19.7	42.9	46.7	5.6	4.8	31.7	42.6	12.7	13.0
Acrida cinerea	34.7	35.2	11.7	18.5	19.8	46.1	19.9	19.8	45.2	45.0	5.6	4.3	33.2	42.1	12.4	12.4
Thrinchus schrenkii	31.6	33.1	14.6	20.7	19.4	44.9	21.0	14.7	40.2	43.0	9.7	7.2	30.4	40.3	15.1	14.2
Physemacris variolosa	36.0	34.1	12.9	17.0	20.2	46.0	19.8	14.0	44.0	39.6	11.1	5.4	33.4	39.9	14.6	12.2
Xyleus modestus	31.7	33.3	13.8	21.1	19.2	45.5	20.4	14.9	41.4	42.8	9.0	6.8	30.8	40.5	14.4	14.3
Mekongiella xizangensis	34.3	34.9	12.6	18.2	19.9	45.6	19.6	14.8	41.4	42.1	9.9	6.5	31.9	40.9	14.1	13.2
Mekongiana xiangchengensis	34.5	35.8	11.8	17.9	21.2	45.7	18.8	14.3	40.7	43.8	8.8	6.8	32.1	41.8	13.1	13.0
Euchorthippus fusigeniculatus	33.2	35.3	12.0	19.6	19.7	45.8	20.2	14.4	43.6	45.7	5.9	4.7	32.2	42.2	12.7	12.9
Gomphocerippus rufus	32.4	35.2	12.4	20.0	19.6	45.6	20.4	14.4	43.1	45.1	6.9	4.9	31.7	42.0	13.2	13.1
Ellipes minuta	30.4	30.6	17.1	22.0	18.8	44.6	20.9	15.7	34.3	36.1	19.1	10.5	27.8	37.1	19.0	16.1
