# Supplementary material for: Complete Mitochondrial Genome of the Free-Living Earwig, Challia fletcheri (Dermaptera: Pygidicranidae) and Phylogeny of Polyneoptera
Source: PLoS One. 2012 Aug 6;7(8):e42056. doi: 10.1371/journal.pone.0042056 (PMC3412835; doi:10.1371/journal.pone.0042056)
Supplement: Table S3 — Composition and skewness of mitochondrial PCGs in Polyneoptera. (PDF) [file pone.0042056.s005.pdf]

Table S3 Composition and skewness of mitochondrial PCGs in Polyneoptera.

Species	Major-strand PCGs			Minor-strand PCGs			Whole PCGs		
	AT%	AT-skewness	GC- skewness	AT%	AT-skewness	GC-skewness	AT%	AT-skewness	GC-skewness
**Dermaptera**									
Challia fletcheri	**68.3**	**-0.327**	**0.383**	**68.9**	**0.059**	**-0.315**	**68.6**	**-0.177**	**0.117**
Plecoptera									
Pteronarcys princeps	68.7	-0.127	-0.168	73.2	-0.259	0.294	70.4	-0.180	-0.006
Mantophasmatodea									
Sclerophasma paresisense	72.5	-0.028	-0.106	76.5	-0.292	0.192	74.0	-0.134	-0.002
Mantodea									
Tamolanica tamolana	72.9	-0.071	-0.167	77.6	-0.240	0.314	74.7	-0.138	-0.004
Blattodea									
Periplaneta fuliginosa	73.1	-0.009	-0.085	76.8	-0.290	0.256	74.5	-0.121	0.035
Blattella germanica	72.3	-0.087	-0.104	77.1	-0.246	0.264	74.2	-0.151	0.021
Eupolyphaga sinensis	69.7	-0.009	-0.168	72.8	-0.322	0.326	70.9	-0.132	0.009
Grylloblattodea									
Grylloblatta sculleni	67.4	-0.039	-0.149	71.8	-0.325	0.227	69.1	-0.154	-0.017
Phasmatodea									
Timema californicum	68.7	-0.053	-0.140	74.0	-0.270	0.234	70.7	-0.141	-0.011
Ramulus hainanense	70.5	0.087	-0.178	73.9	-0.389	0.278	71.6	-0.106	-0.021
Phraortes sp. Iriomote	75.9	0.082	-0.146	79.3	-0.353	0.234	77.2	-0.092	-0.011
Ramulus irregulariterdentatus	74.0	0.040	-0.099	77.9	-0.323	0.177	75.5	-0.105	-0.002
Phraortes illepidus	75.2	0.082	-0.137	78.7	-0.375	0.225	76.6	-0.101	-0.009
Entoria okinawaensis	74.0	0.051	-0.148	78.2	-0.345	0.237	75.6	-0.108	-0.015
Megacrania alpheus adan	74.3	0.110	-0.185	77.3	-0.376	0.302	75.5	-0.083	-0.011
Heteropteryx dilatata	74.3	0.092	-0.177	77.5	-0.382	0.274	75.6	-0.098	-0.017
Phobaeticus serratipes	74.9	0.105	-0.184	77.4	-0.389	0.303	75.9	-0.091	-0.007
Micadina phluctainoides	75.5	0.058	-0.081	78.8	-0.336	0.155	76.8	-0.099	-0.002
Isoptera									

Reticulitermes santonensis	63.0	0.186	-0.266	67.1	-0.532	0.353	64.5	-0.099	-0.045
Reticulitermes hageni	62.5	0.186	-0.261	65.7	-0.534	0.334	63.7	-0.099	-0.044
Reticulitermes virginicus	63.0	0.187	-0.268	67.4	-0.514	0.347	64.7	-0.094	-0.050
Reticulitermes flavipes	63.1	0.188	-0.269	67.3	-0.521	0.349	64.7	-0.096	0.303
Orthoptera									
Deracantha onos	65.9	-0.093	-0.266	70.7	-0.227	0.319	67.7	-0.145	-0.068
Troglophilus neglectus	70.6	-0.121	-0.206	75.5	-0.213	0.285	72.5	-0.158	-0.039
Anabrus simplex	65.7	-0.114	-0.253	70.7	-0.255	0.318	67.6	-0.170	-0.054
Gampsocleis gratiosa	61.1	-0.074	-0.292	67.5	-0.283	0.334	63.6	-0.159	-0.077
Ruspolia dubia	68.5	-0.121	-0.202	72.2	-0.233	0.297	69.9	-0.166	-0.024
Myrmecophilus manni	67.7	-0.057	-0.292	71.0	-0.259	0.330	69.0	-0.138	-0.067
Gryllotalpa orientalis	67.9	-0.099	-0.277	71.8	-0.254	0.392	69.4	-0.160	-0.043
Teleogryllus emma	71.1	-0.033	-0.208	74.8	-0.314	0.358	72.6	-0.145	-0.007
Gryllotalpa pluvialis	69.4	-0.104	-0.273	73.9	-0.240	0.418	71.2	-0.158	-0.031
Elimaea cheni	69.9	-0.119	-0.213	73.9	-0.202	-0.351	71.5	-0.152	-0.013
Acrida willemsei	73.9	0.044	-0.114	77.7	-0.370	0.218	75.4	-0.120	0.002
Schistocerca gregaria	70.4	0.017	-0.141	75.1	-0.373	0.219	72.2	-0.139	-0.018
Gomphocerus licenti	73.4	-0.006	-0.091	75.6	-0.382	0.066	74.3	-0.153	0.017
Arcyptera coreana	74.4	0.002	-0.093	78.0	-0.329	0.172	75.8	-0.129	0.000
Chorthippus chinensis	73.8	0.008	-0.117	76.8	-0.358	0.200	74.9	-0.137	-0.004
Phlaeoba albonema	72.0	0.025	-0.118	75.9	-0.370	0.234	73.5	-0.132	0.005
Oxya chinensis	73.7	-0.022	-0.083	77.5	-0.346	0.197	75.2	-0.150	0.014
Calliptamus italicus	70.8	0.004	-0.131	75.0	-0.356	0.221	72.4	-0.139	-0.008
Prumna arctica	73.4	-0.002	-0.112	78.0	-0.347	0.163	75.2	-0.139	-0.018
Traulia szetschuanensis	72.4	0.011	-0.117	76.3	-0.359	0.219	73.9	-0.135	0.000
Ognevia longipennis	73.8	-0.019	-0.101	77.6	-0.330	0.219	75.2	-0.142	0.010
Oedaleus decorus asiaticus	72.2	0.093	-0.172	76.6	-0.387	0.198	73.9	-0.098	-0.041
Gastrimargus marmoratus	73.4	-0.006	-0.091	75.6	-0.382	0.204	73.9	-0.153	0.017
Locusta migratoria migratoria	72.6	0.065	-0.167	76.7	-0.385	0.174	74.2	-0.114	-0.045
Locusta migratoria	72.6	0.068	-0.168	76.5	-0.389	0.199	74.1	-0.113	-0.040

Locusta migratoria tibetensis	72.7	0.065	-0.168	76.6	-0.381	0.192	74.2	-0.112	-0.043
Locusta migratoria manilensis	72.6	0.067	-0.166	76.5	-0.383	0.195	74.1	-0.112	-0.040
Atractomorpha sinensis	72.6	0.026	-0.129	75.1	-0.393	0.244	73.6	-0.137	0.005
Gomphocerus sibiricus	72.8	-0.043	-0.091	76.6	-0.363	0.204	74.3	-0.146	0.012
Acrida cinerea	73.8	0.047	-0.111	77.7	-0.368	0.205	75.3	-0.118	-0.001
Thrinchus schrenkii	69.1	0.025	-0.187	73.3	-0.391	0.261	70.7	-0.141	-0.030
Physemacris variolosa	71.6	0.067	-0.254	75.9	-0.325	0.218	73.3	-0.089	-0.090
Xyleus modestus	70.0	0.043	-0.160	73.3	-0.412	0.274	71.3	-0.137	-0.005
Mekongiella xizangensis	71.7	0.032	-0.191	74.5	-0.364	0.252	72.8	-0.123	-0.033
Mekongiana xiangchengensis	72.7	0.018	-0.148	75.8	-0.360	0.251	73.9	-0.131	-0.006
Euchorthippus fusigeniculatus	73.1	0.003	-0.101	76.5	-0.348	0.206	74.4	-0.135	0.007
Gomphocerippus rufus	72.0	0.009	-0.121	76.2	-0.365	0.217	73.7	-0.139	-0.004
Ellipes minuta	63.6	-0.101	-0.171	66.9	-0.207	0.286	64.9	-0.143	-0.084
