# Supplementary material for: Complete Mitochondrial Genome of the Free-Living Earwig, Challia fletcheri (Dermaptera: Pygidicranidae) and Phylogeny of Polyneoptera
Source: PLoS One. 2012 Aug 6;7(8):e42056. doi: 10.1371/journal.pone.0042056 (PMC3412835; doi:10.1371/journal.pone.0042056)
Supplement: Table S4 — Results of topological tests for 2 datasets, showing values from 6 statistical tests performed. (PDF) [file pone.0042056.s006.pdf]

Table S4 Results of topological tests for two datasets, showing values from six statistical tests performed.

tests	dataset	
	**PCG123RNA**	**PCG12RNA**
ELW	0.6871765	0.9225586
BP	0.6874	0.92278
KH	1	1
SH	1	1
WSH	1	1
AU	0.6813492	0.925226

The full topology obtained from the dataset PCG123RNA is presented in Figure 7A, while that from PCG12RNA is presented in Figure 7B.
